# Supplementary figures and images for: Personalized expression of bitter ‘taste’ receptors in human skin
Source: PLoS One. 2018 Oct 17;13(10):e0205322. doi: 10.1371/journal.pone.0205322 (PMC6192714; doi:10.1371/journal.pone.0205322)

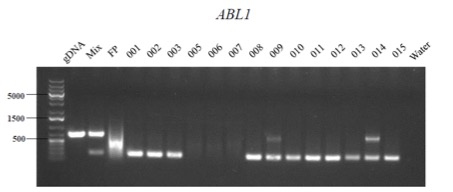

Supplement: S1 Fig — PCR was performed with genomic DNA from skin (gDNA), a mixture of genomic DNA and cDNA from skin (Mix), cDNA from fungiform papillae (FP), and cDNA from 14 skin samples (001–015). Water was used as a no-template control. The larger band at 793 base pairs (bp) includes introns, and the smaller band at 293 bp does not contain introns. Genomic DNA was used as a positive control for the larger band size. A mix was used as a positive control for both bands. The smear at FP is likely caused by nonspecific binding. (JPG) [file pone.0205322.s001.jpg]

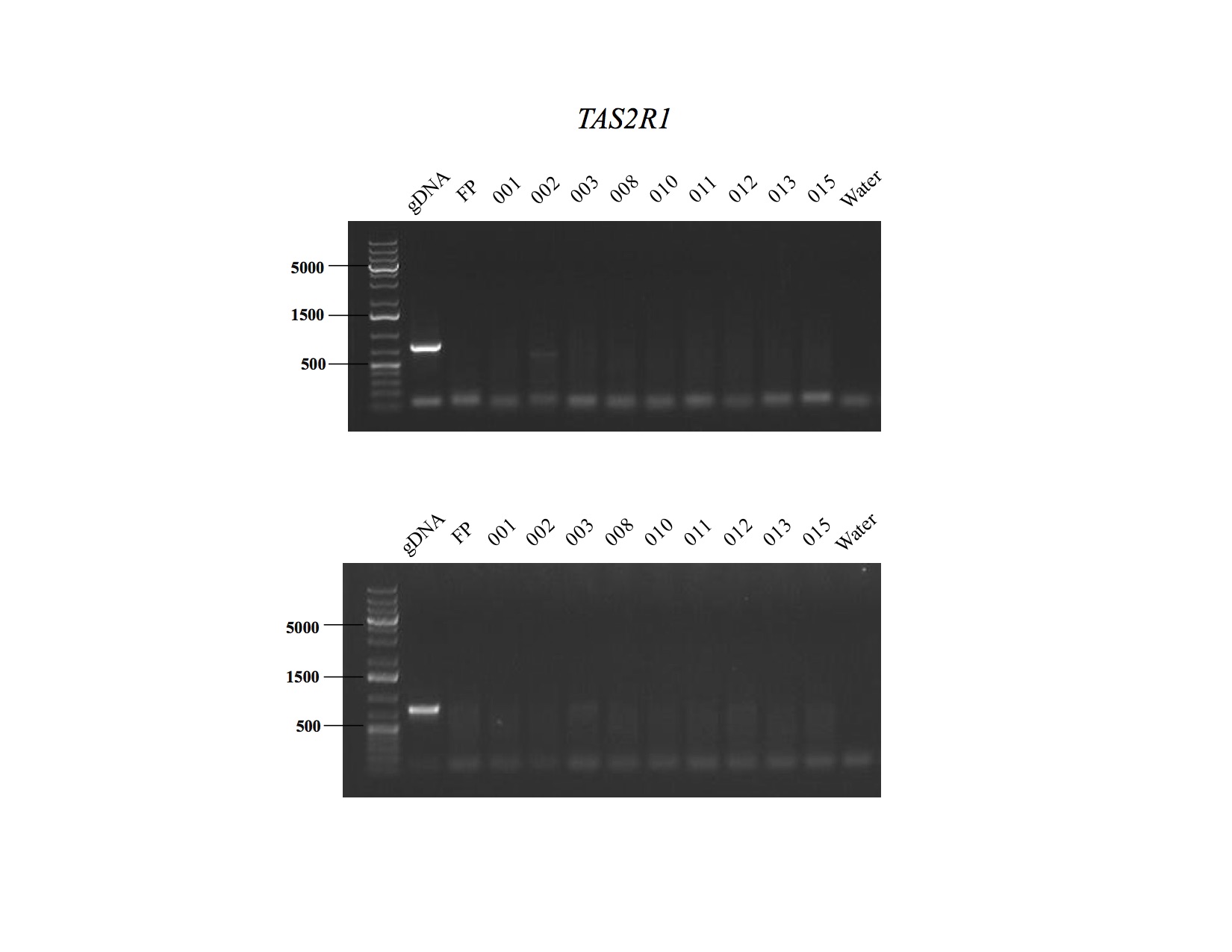

Supplement: S2 Fig — PCR was performed with genomic DNA from skin (gDNA), cDNA from fungiform papillae (FP), and cDNA from nine skin samples. Water was used as a no-template control. The expected band size is 813 bp. The experiment was replicated (bottom panel) because taste receptors are not abundant and can have variable results. (JPG) [file pone.0205322.s002.jpg]

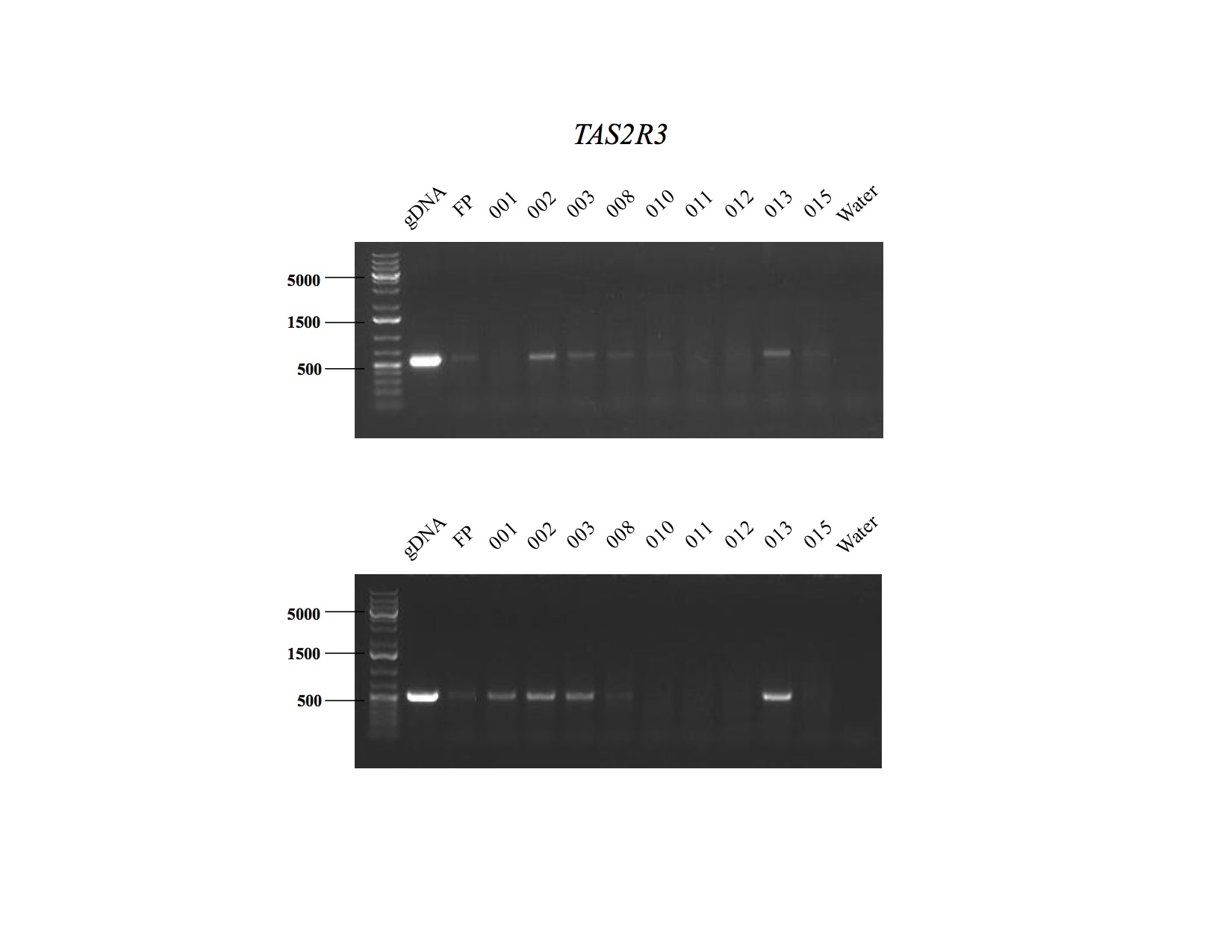

Supplement: S3 Fig — PCR was performed with genomic DNA from skin (gDNA), cDNA from fungiform papillae (FP), and cDNA from nine skin samples. Water was used as a no-template control. The expected band size is 575 bp. The experiment was replicated (bottom panel) because taste receptors are not abundant and can have variable results. (JPG) [file pone.0205322.s003.jpg]

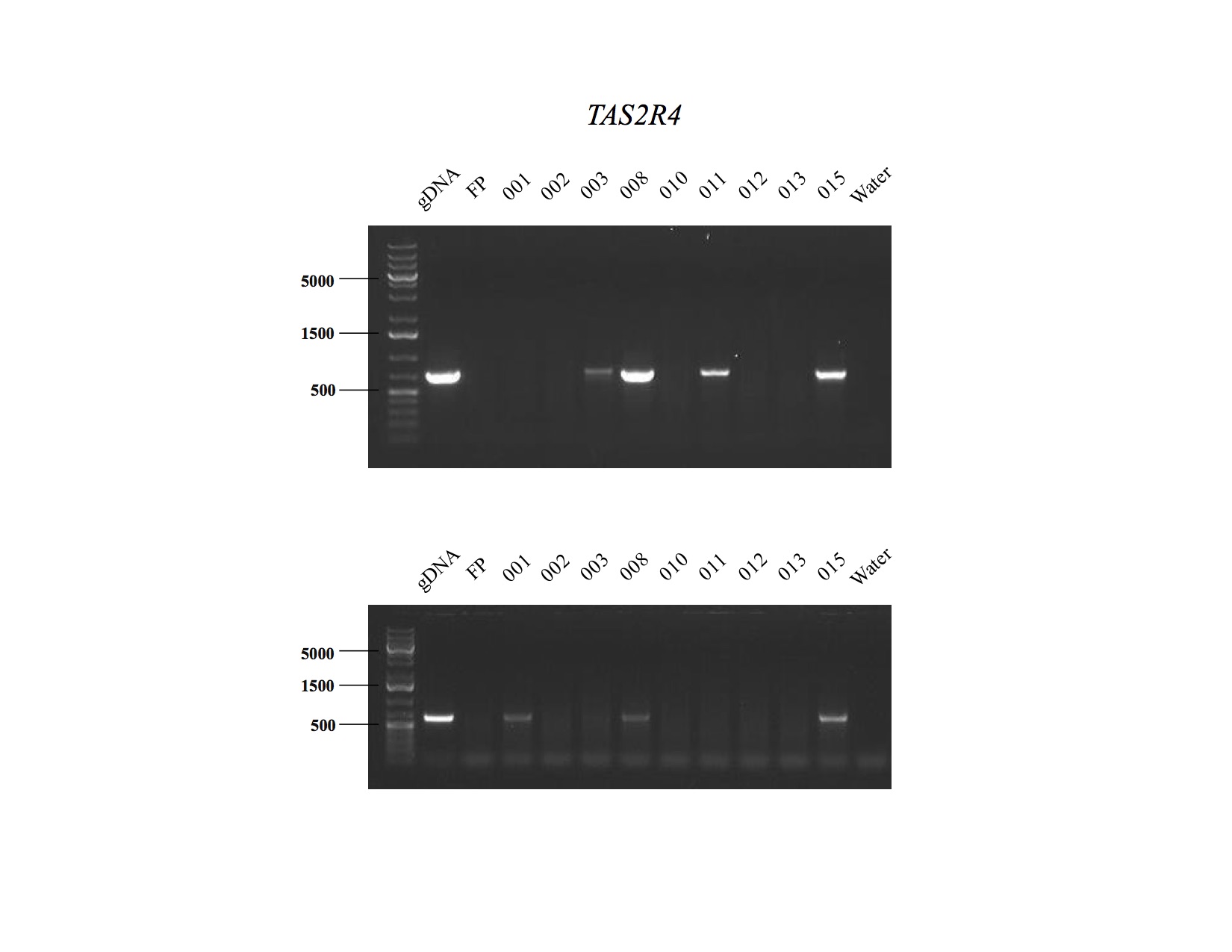

Supplement: S4 Fig — PCR was performed with genomic DNA from skin (gDNA), cDNA from fungiform papillae (FP), and cDNA from nine skin samples. Water was used as a no-template control. The expected band size is 749 bp. The experiment was replicated (bottom panel) because taste receptors are not abundant and can have variable results. (JPG) [file pone.0205322.s004.jpg]

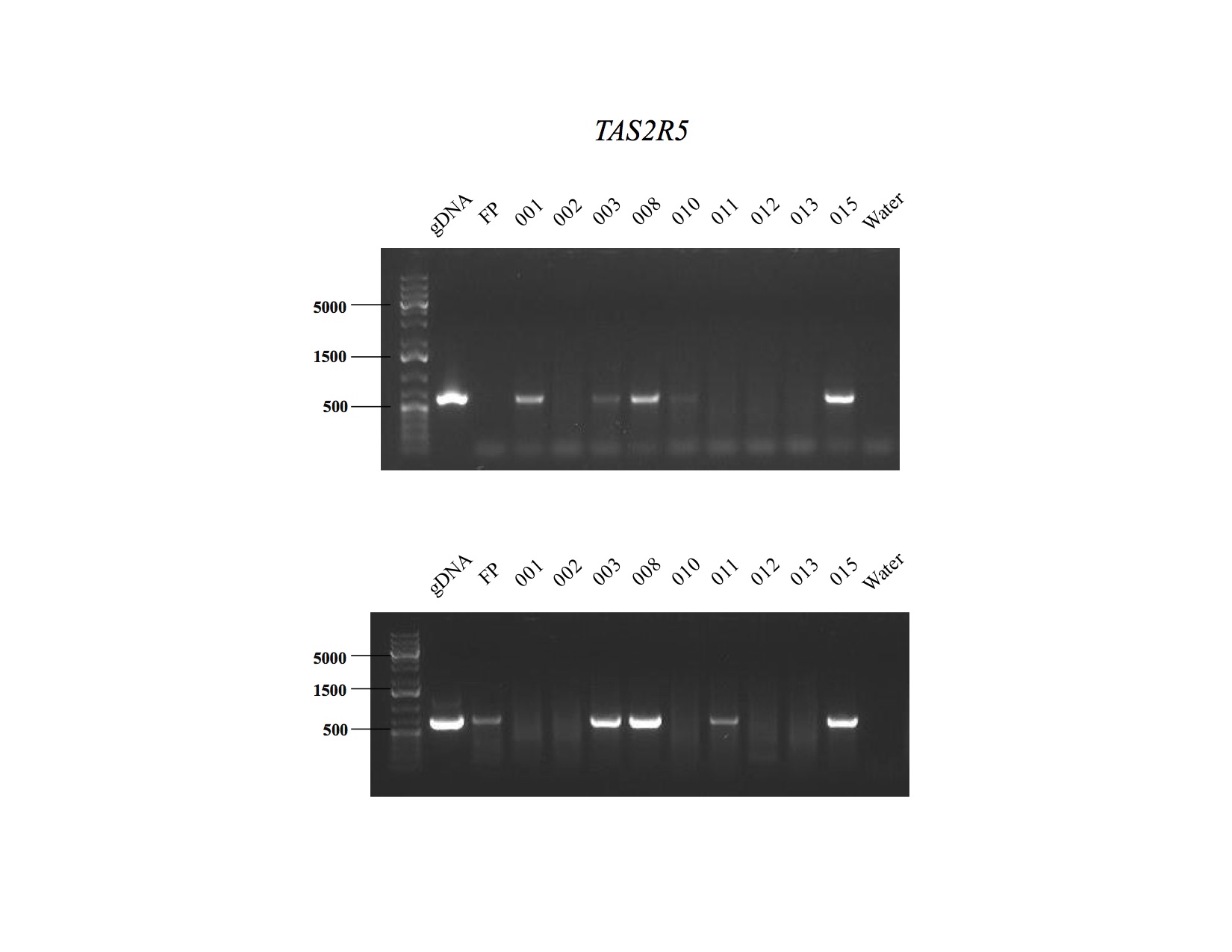

Supplement: S5 Fig — PCR was performed with genomic DNA from skin (gDNA), cDNA from fungiform papillae (FP), and cDNA from nine skin samples. Water was used as a no-template control. The expected band size is 667 bp. The experiment was replicated (bottom panel) because taste receptors are not abundant and can have variable results. (JPG) [file pone.0205322.s005.jpg]

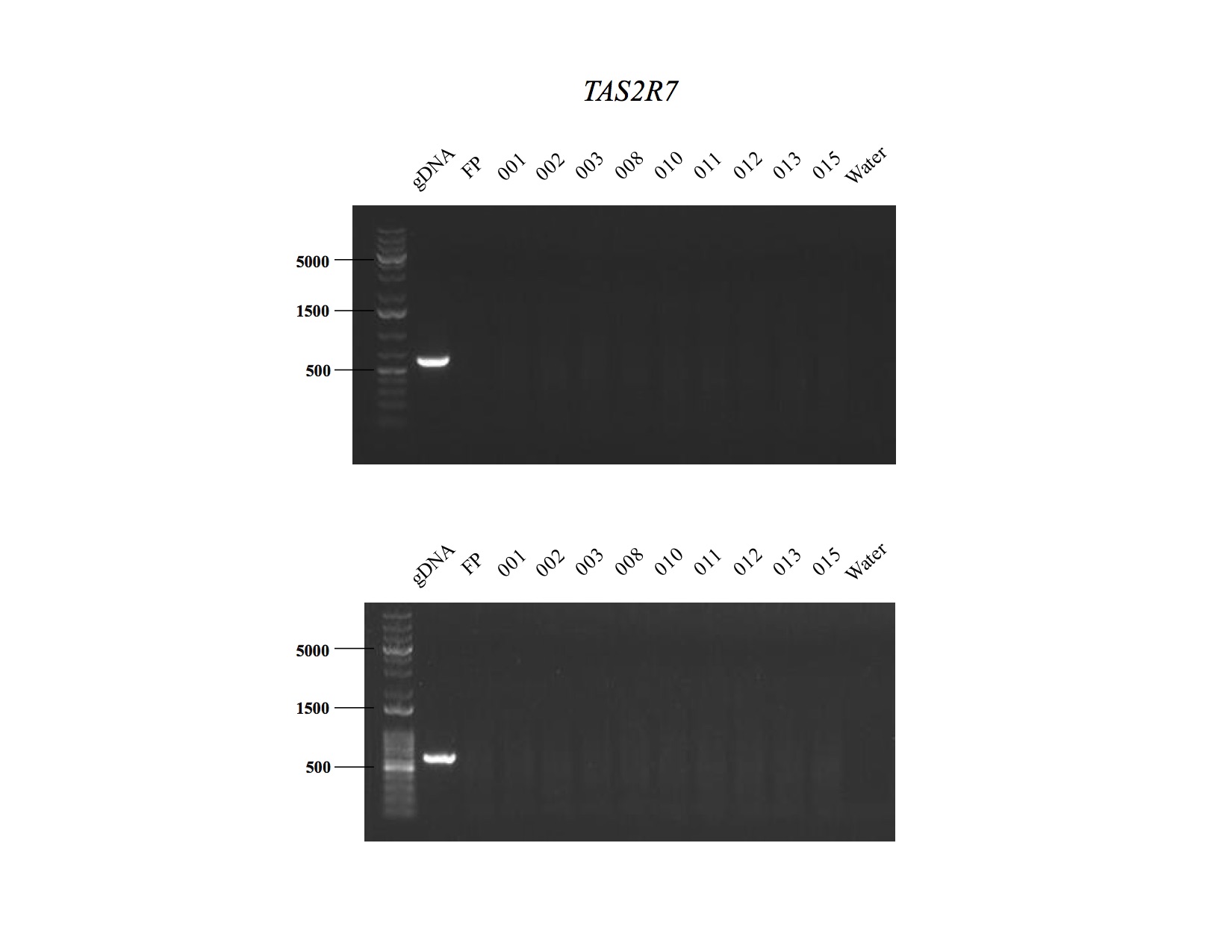

Supplement: S6 Fig — PCR was performed with genomic DNA from skin (gDNA), cDNA from fungiform papillae (FP), and cDNA from nine skin samples. Water was used as a no-template control. The expected band size is 658 bp. The experiment was replicated (bottom panel) because taste receptors are not abundant and can have variable results. (JPG) [file pone.0205322.s006.jpg]

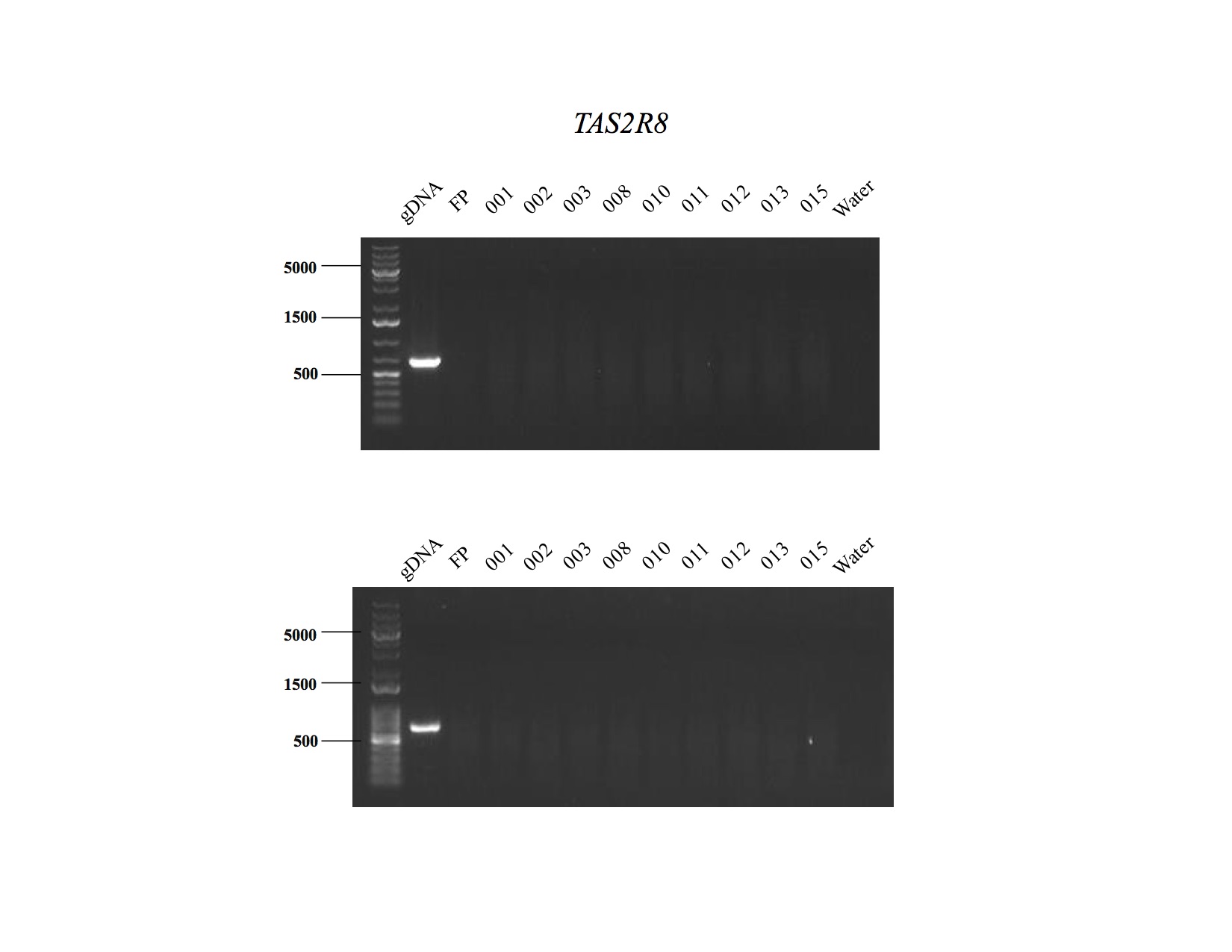

Supplement: S7 Fig — PCR was performed with genomic DNA from skin (gDNA), cDNA from fungiform papillae (FP), and cDNA from nine skin samples. Water was used as a no-template control. The expected band size is 723 bp. The experiment was replicated (bottom panel) because taste receptors are not abundant and can have variable results. (JPG) [file pone.0205322.s007.jpg]

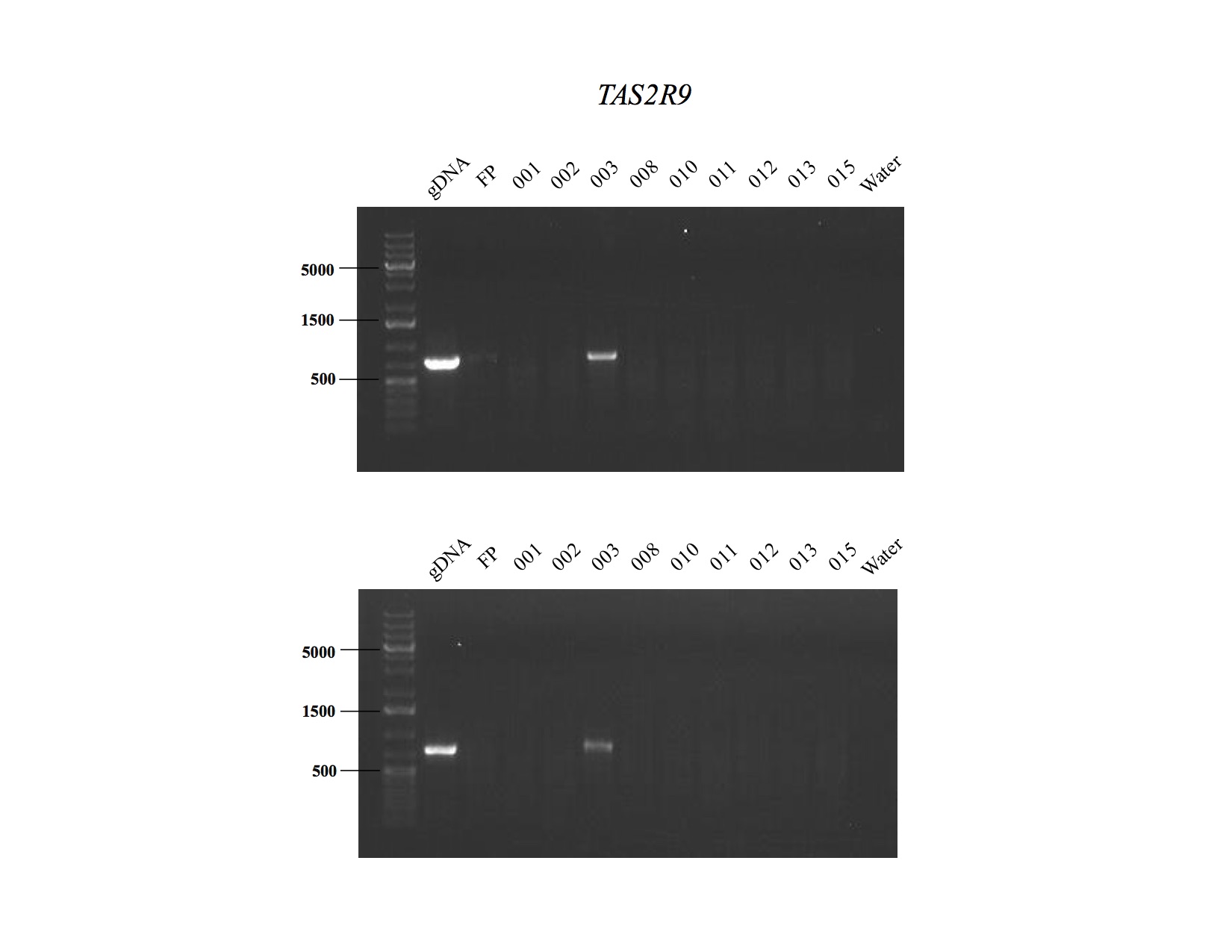

Supplement: S8 Fig — PCR was performed with genomic DNA from skin (gDNA), cDNA from fungiform papillae (FP), and cDNA from nine skin samples. Water was used as a no-template control. The expected band size is 807 bp. The experiment was replicated (bottom panel) because taste receptors are not abundant and can have variable results. (JPG) [file pone.0205322.s008.jpg]

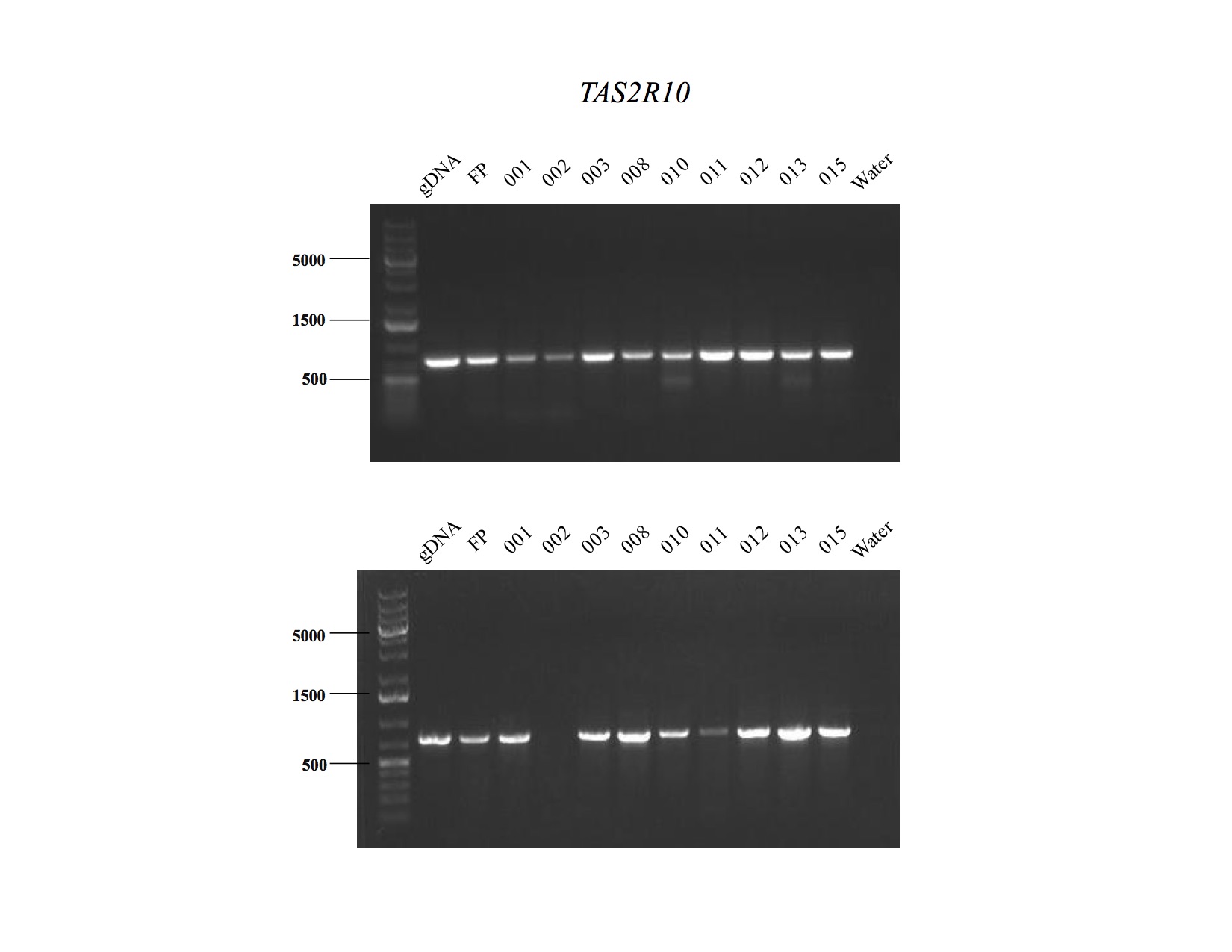

Supplement: S9 Fig — PCR was performed with genomic DNA from skin (gDNA), cDNA from fungiform papillae (FP), and cDNA from nine skin samples. Water was used as a no-template control. The expected band size is 783 bp. The experiment was replicated (bottom panel) because taste receptors are not abundant and can have variable results. (JPG) [file pone.0205322.s009.jpg]

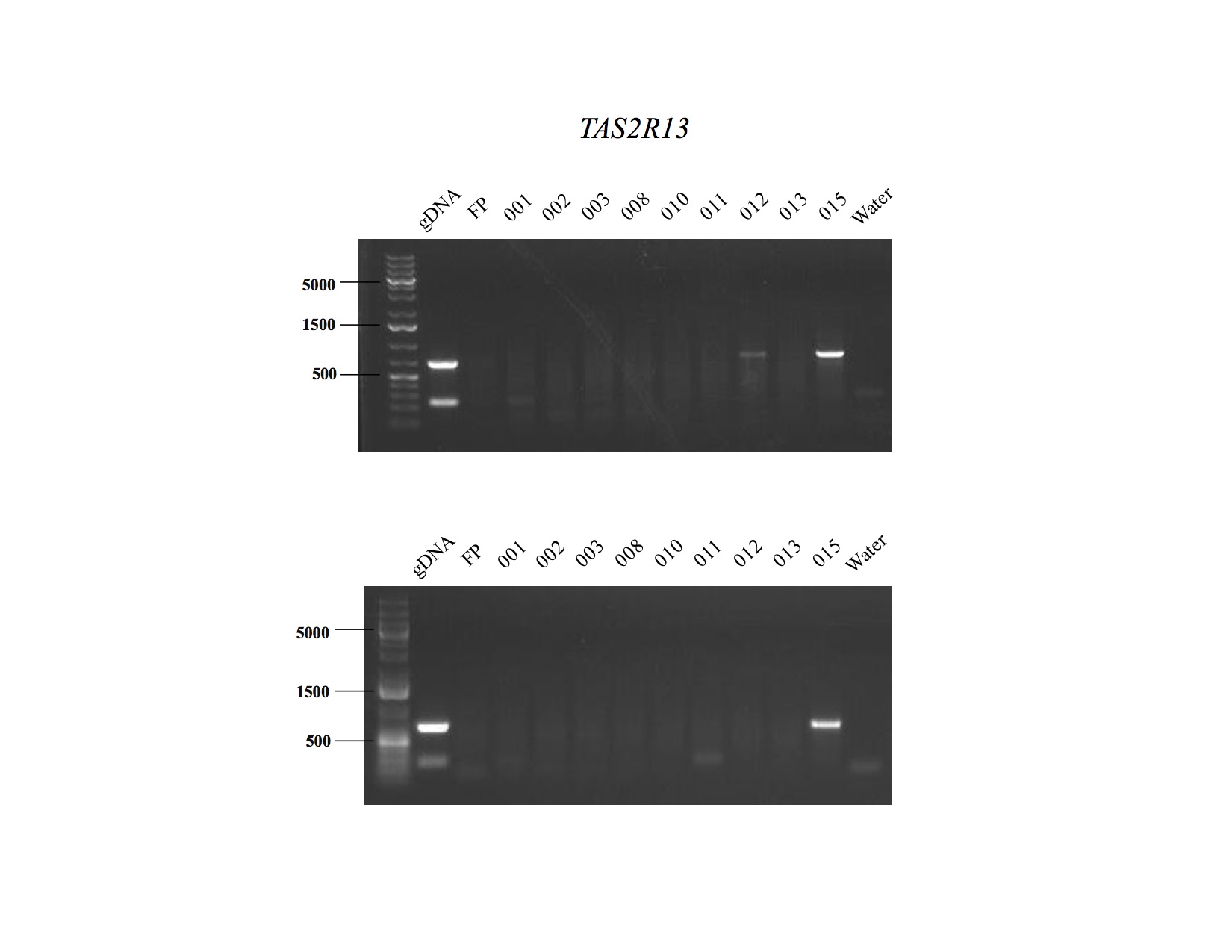

Supplement: S10 Fig — PCR was performed with genomic DNA from skin (gDNA), cDNA from fungiform papillae (FP), and cDNA from nine skin samples. Water was used as a no-template control. The expected band size is 742 bp. The experiment was replicated (bottom panel) because taste receptors are not abundant and can have variable results. (JPG) [file pone.0205322.s010.jpg]

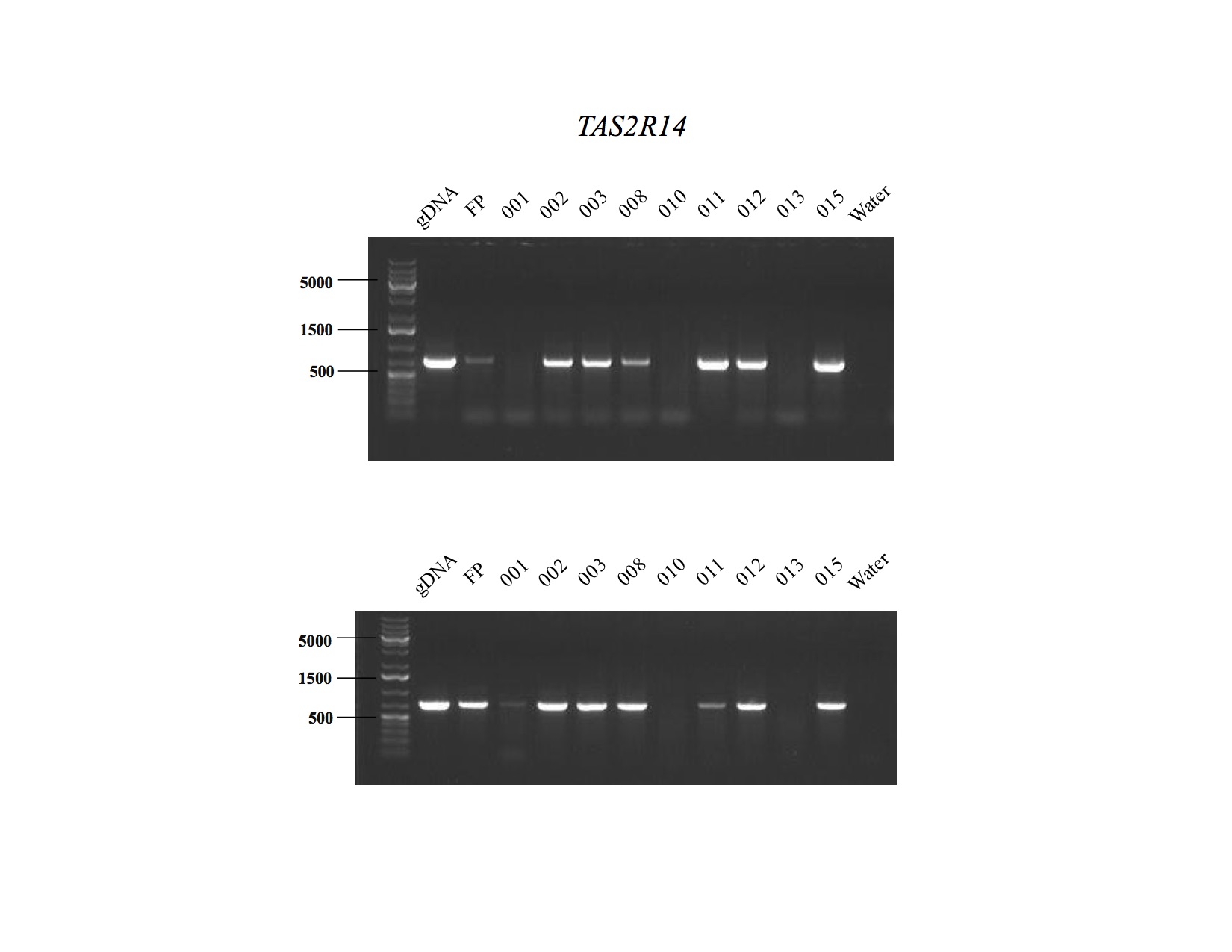

Supplement: S11 Fig — PCR was performed with genomic DNA from skin (gDNA), cDNA from fungiform papillae (FP), and cDNA from nine skin samples. Water was used as a no-template control. The expected band size is 796 bp. The experiment was replicated (bottom panel) because taste receptors are not abundant and can have variable results. (JPG) [file pone.0205322.s011.jpg]

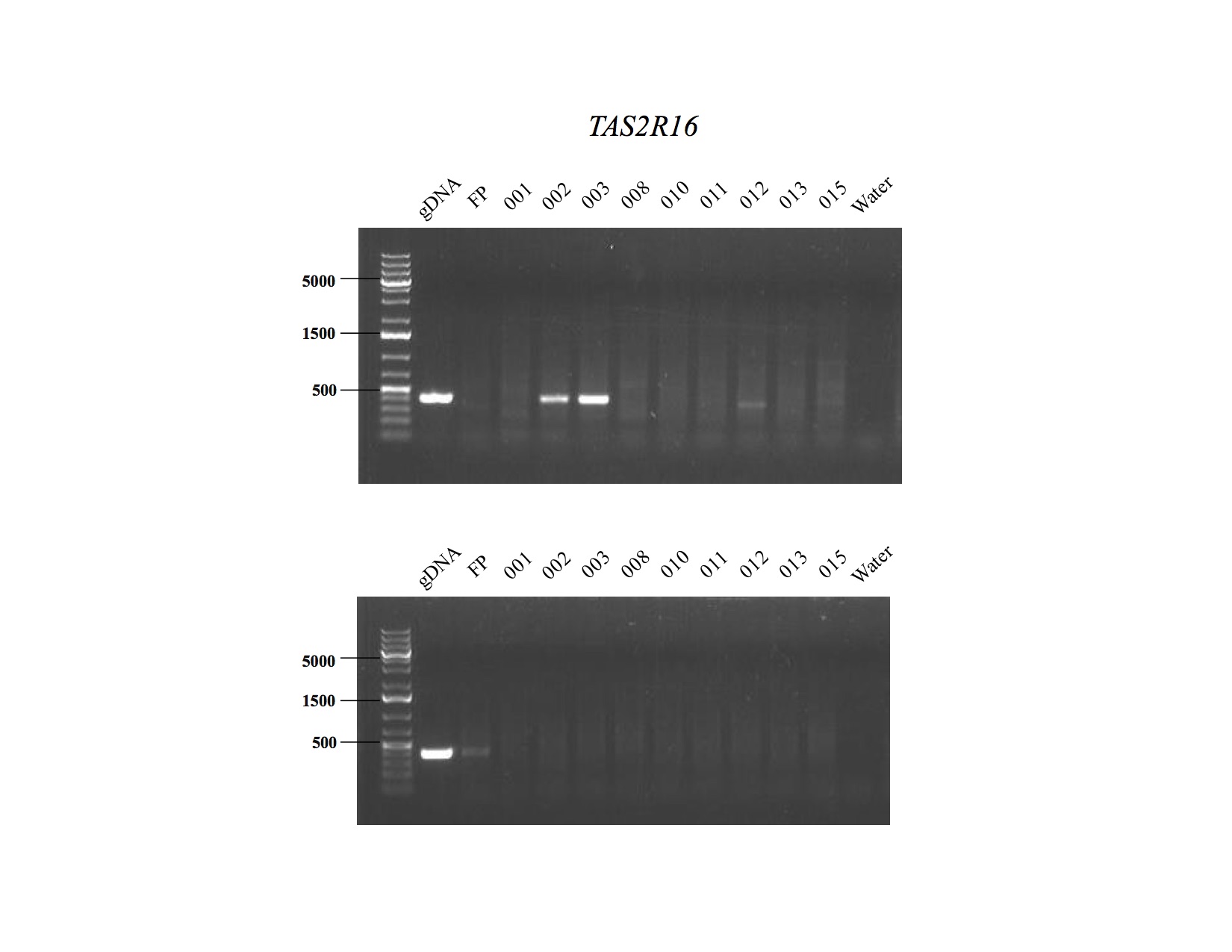

Supplement: S12 Fig — PCR was performed with genomic DNA from skin (gDNA), cDNA from fungiform papillae (FP), and cDNA from nine skin samples. Water was used as a no-template control. The expected band size is 419 bp. The experiment was replicated (bottom panel) because taste receptors are not abundant and can have variable results. (JPG) [file pone.0205322.s012.jpg]

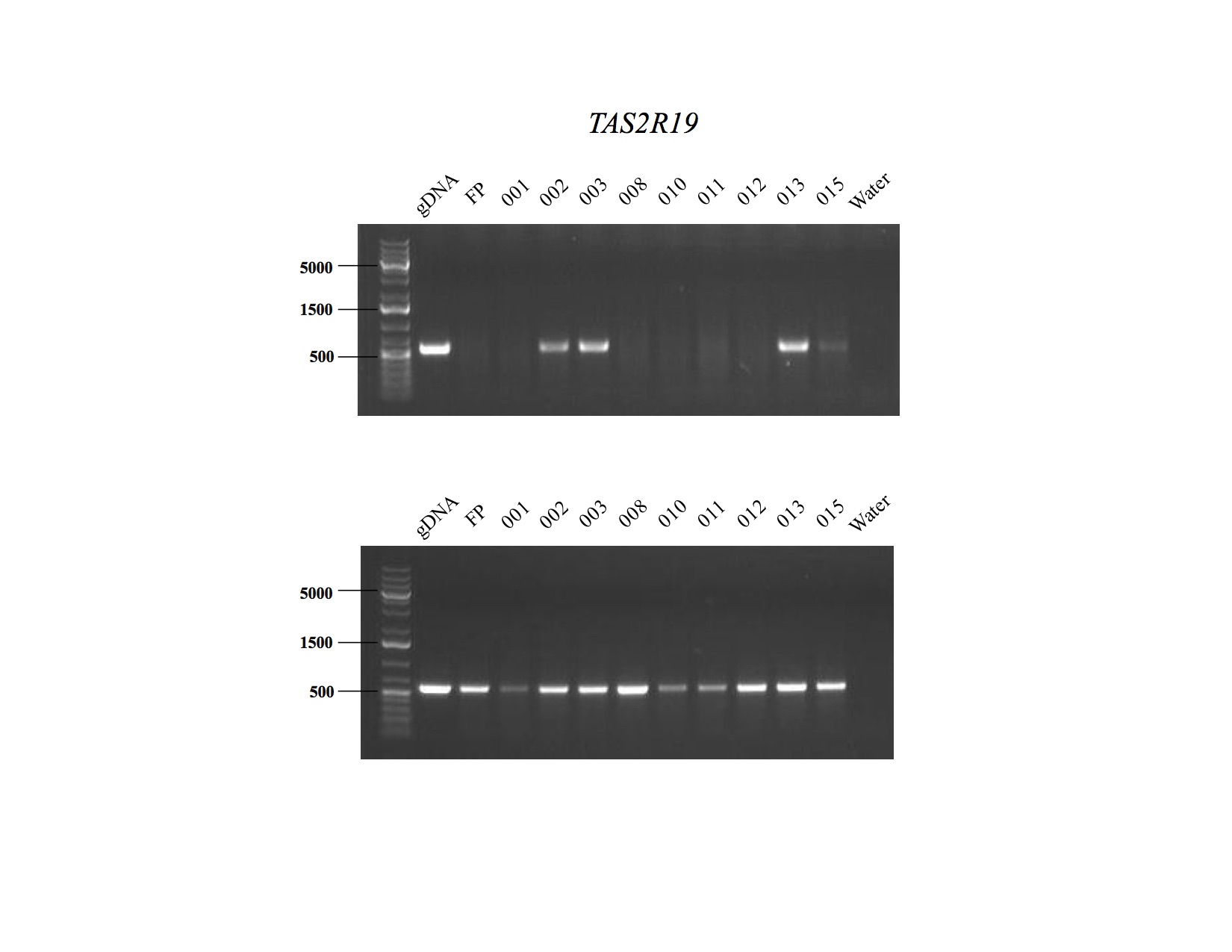

Supplement: S13 Fig — PCR was performed with genomic DNA from skin (gDNA), cDNA from fungiform papillae (FP), and cDNA from nine skin samples. Water was used as a no-template control. The expected band size is 606 bp. The experiment was replicated (bottom panel) because taste receptors are not abundant and can have variable results. (JPG) [file pone.0205322.s013.jpg]

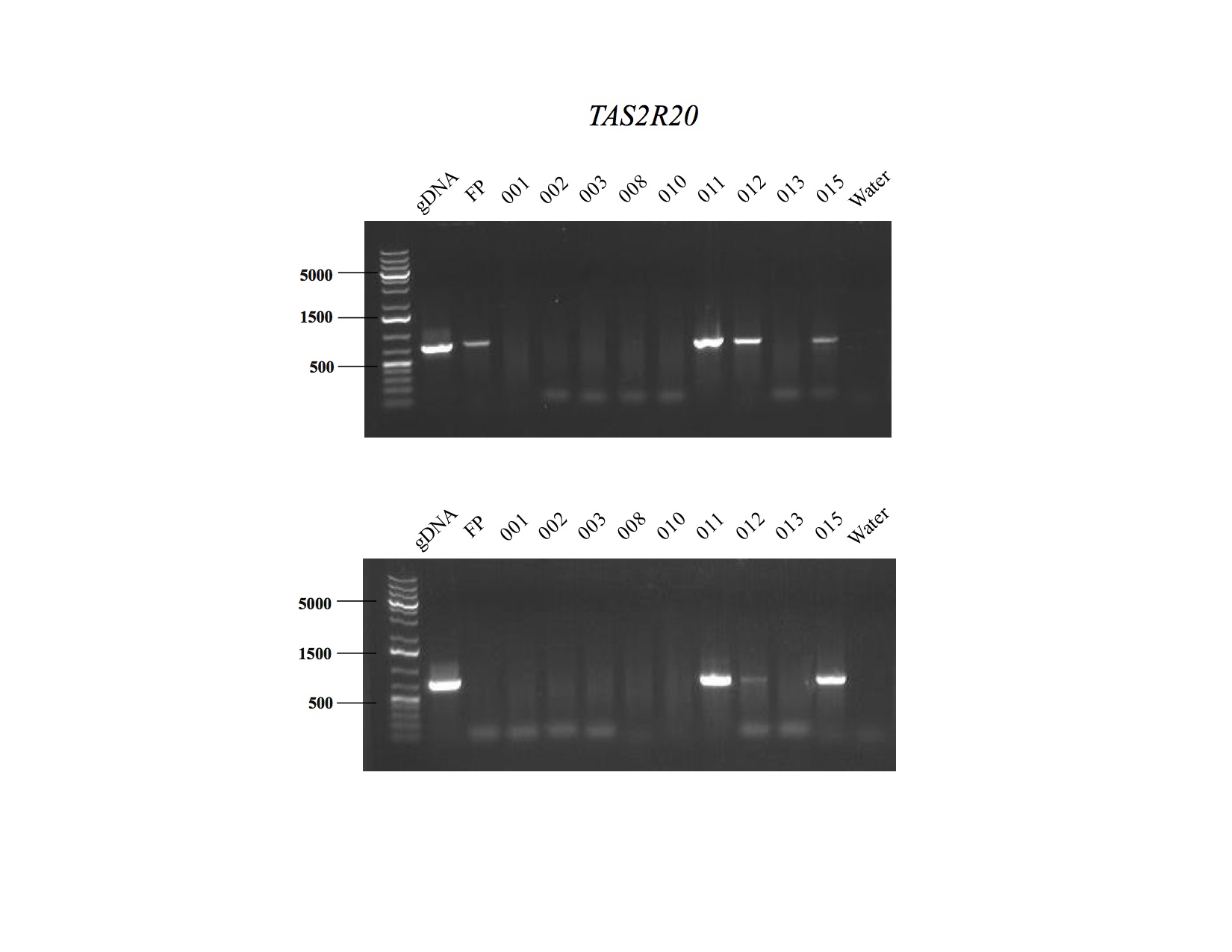

Supplement: S14 Fig — PCR was performed with genomic DNA from skin (gDNA), cDNA from fungiform papillae (FP), and cDNA from nine skin samples. Water was used as a no-template control. The expected band size is 770 bp. The experiment was replicated (bottom panel) because taste receptors are not abundant and can have variable results. (JPG) [file pone.0205322.s014.jpg]

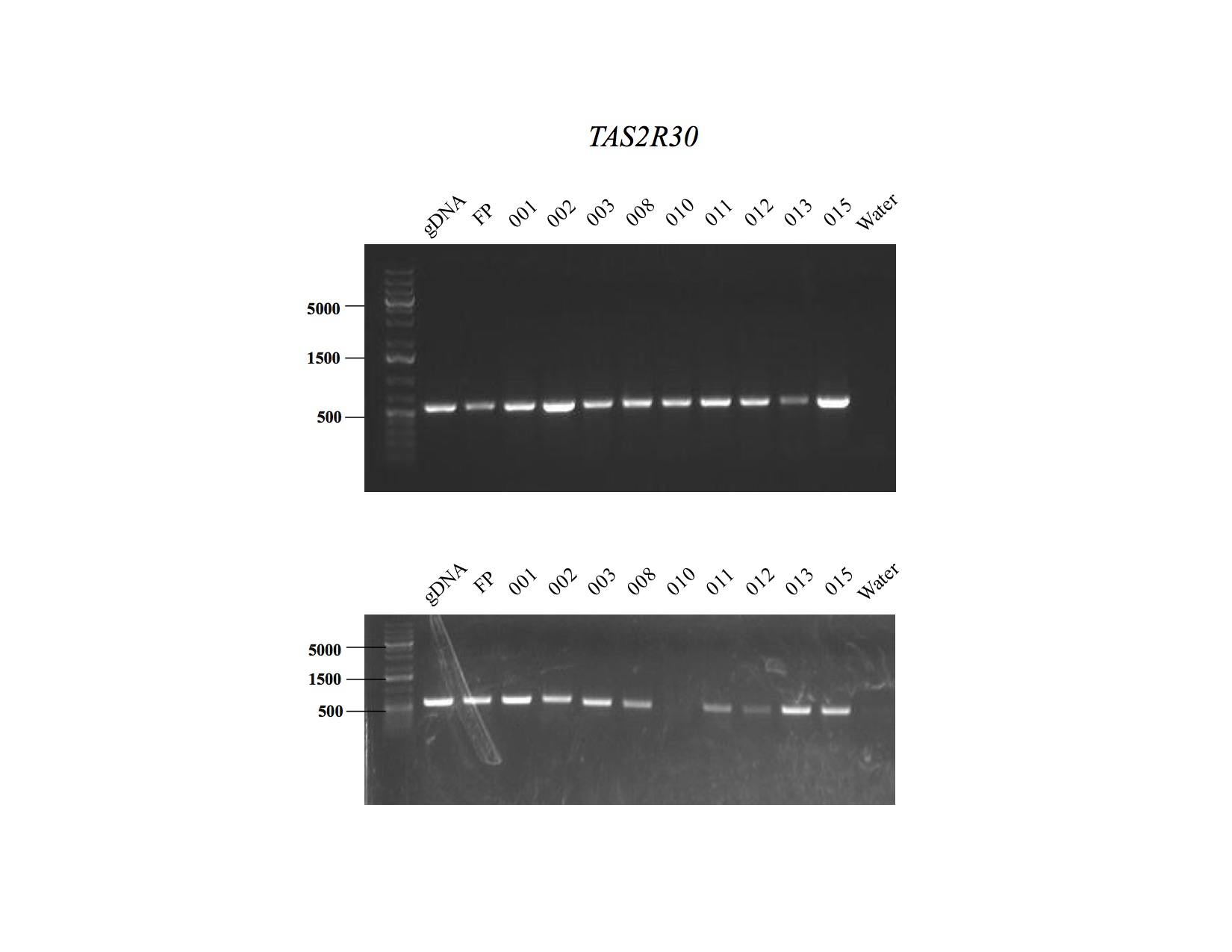

Supplement: S15 Fig — PCR was performed with genomic DNA from skin (gDNA), cDNA from fungiform papillae (FP), and cDNA from nine skin samples. Water was used as a no-template control. The expected band size is 603 bp. The experiment was replicated (bottom panel) because taste receptors are not abundant and can have variable results. (JPG) [file pone.0205322.s015.jpg]

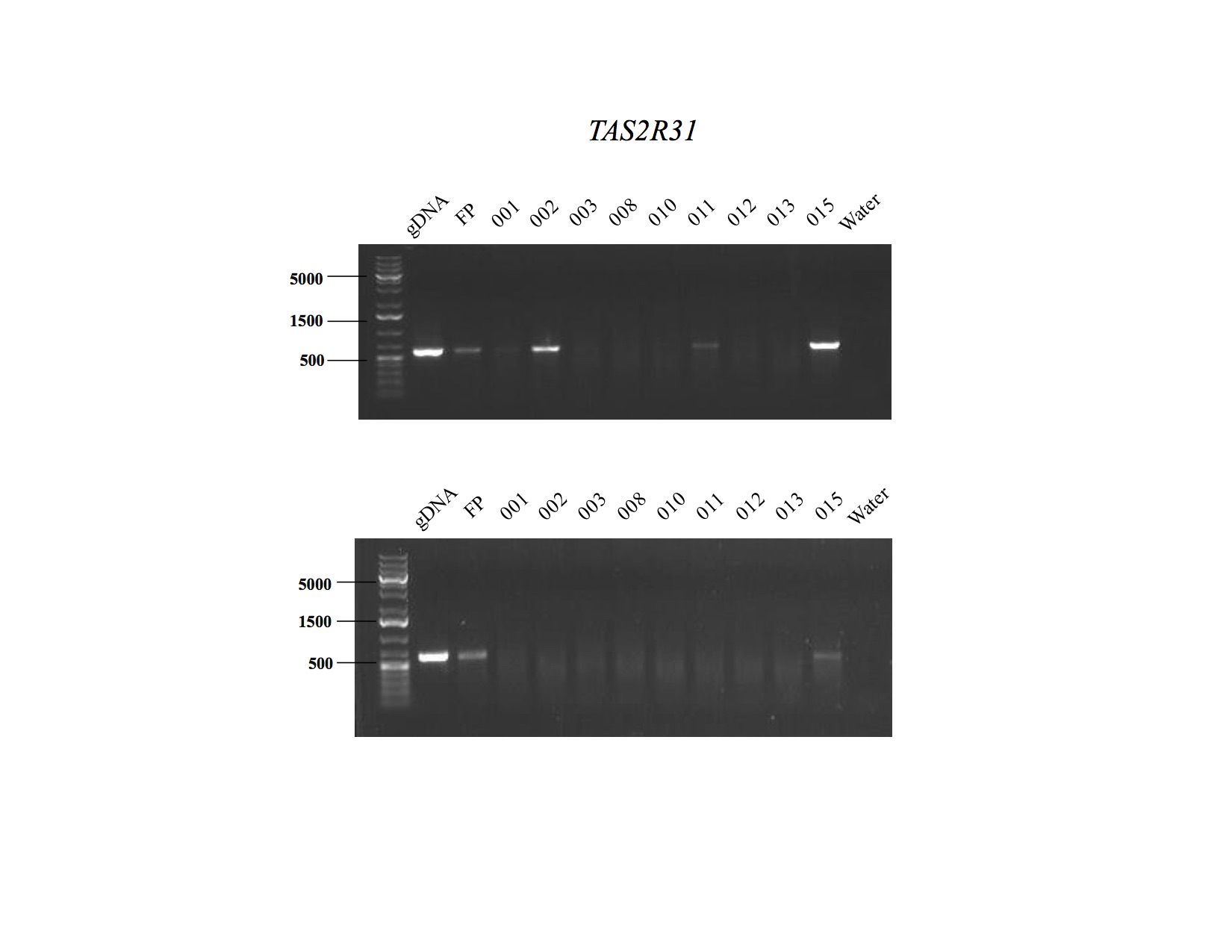

Supplement: S16 Fig — PCR was performed with genomic DNA from skin (gDNA), cDNA from fungiform papillae (FP), and cDNA from nine skin samples. Water was used as a no-template control. The expected band size is 661 bp. The experiment was replicated (bottom panel) because taste receptors are not abundant and can have variable results. (JPG) [file pone.0205322.s016.jpg]

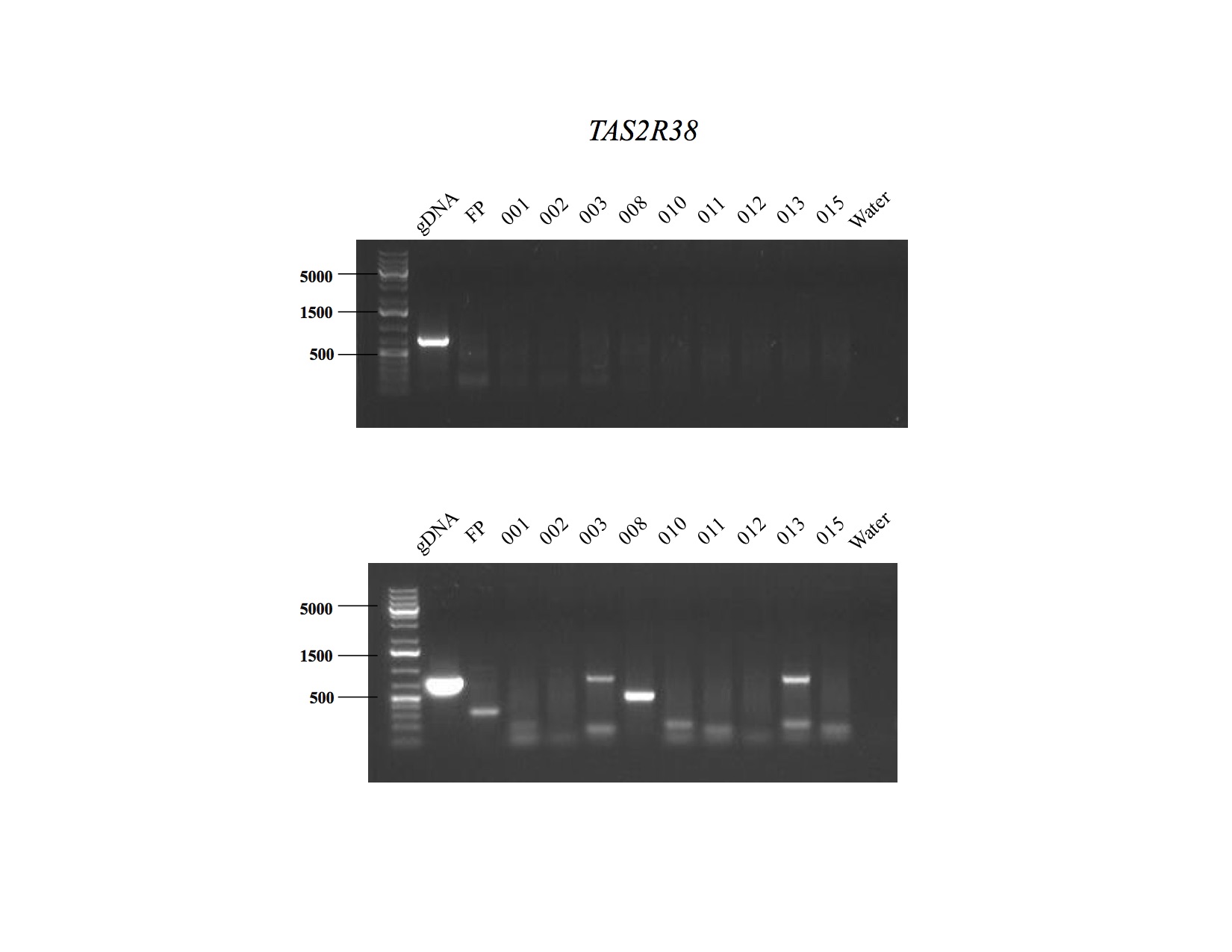

Supplement: S17 Fig — PCR was performed with genomic DNA from skin (gDNA), cDNA from fungiform papillae (FP), and cDNA from nine skin samples. Water was used as a no-template control. The expected band size is 766 bp. Multiple bands are likely because of non-specific binding. The experiment was replicated (bottom panel) because taste receptors are not abundant and can have variable results. (JPG) [file pone.0205322.s017.jpg]

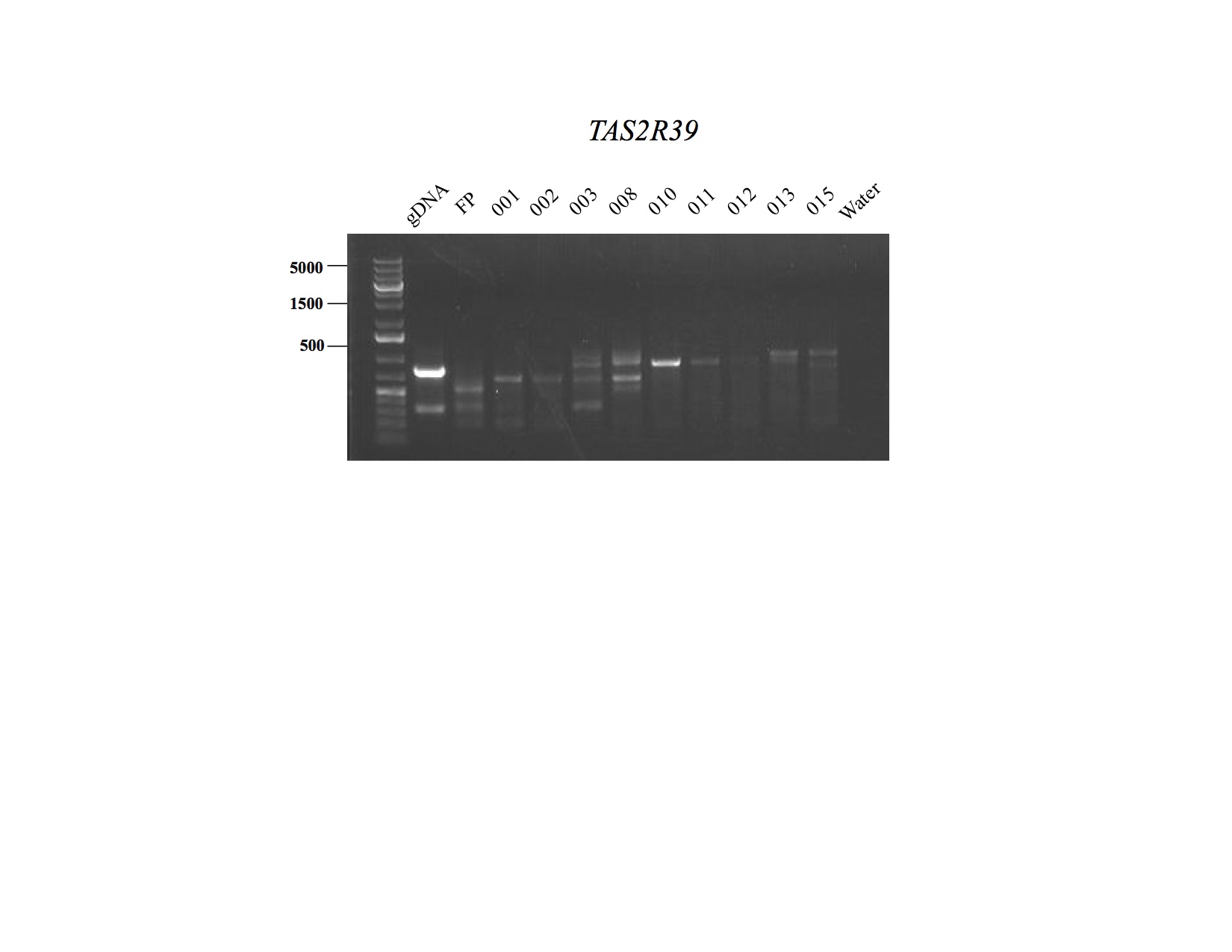

Supplement: S18 Fig — PCR was performed with genomic DNA from skin (gDNA), cDNA from fungiform papillae (FP), and cDNA from nine skin samples. Water was used as a no-template control. The expected band size is 841 bp. The experiment was replicated, but results were omitted because of non-specific binding. (JPG) [file pone.0205322.s018.jpg]

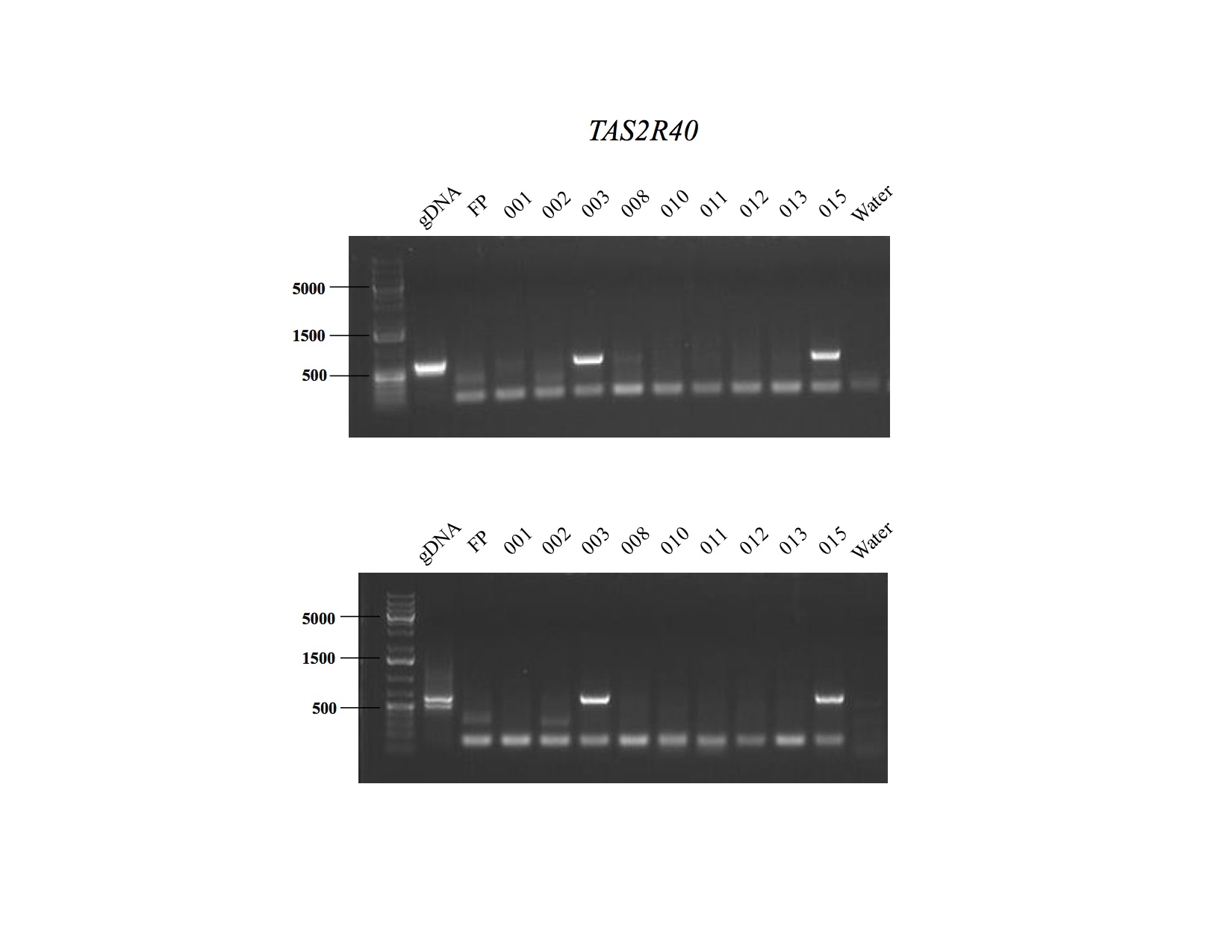

Supplement: S19 Fig — PCR was performed with genomic DNA from skin (gDNA), cDNA from fungiform papillae (FP), and cDNA from nine skin samples. Water was used as a no-template control. The expected band size is 685 bp. The experiment was replicated (bottom panel) because taste receptors are not abundant and can have variable results. (JPG) [file pone.0205322.s019.jpg]

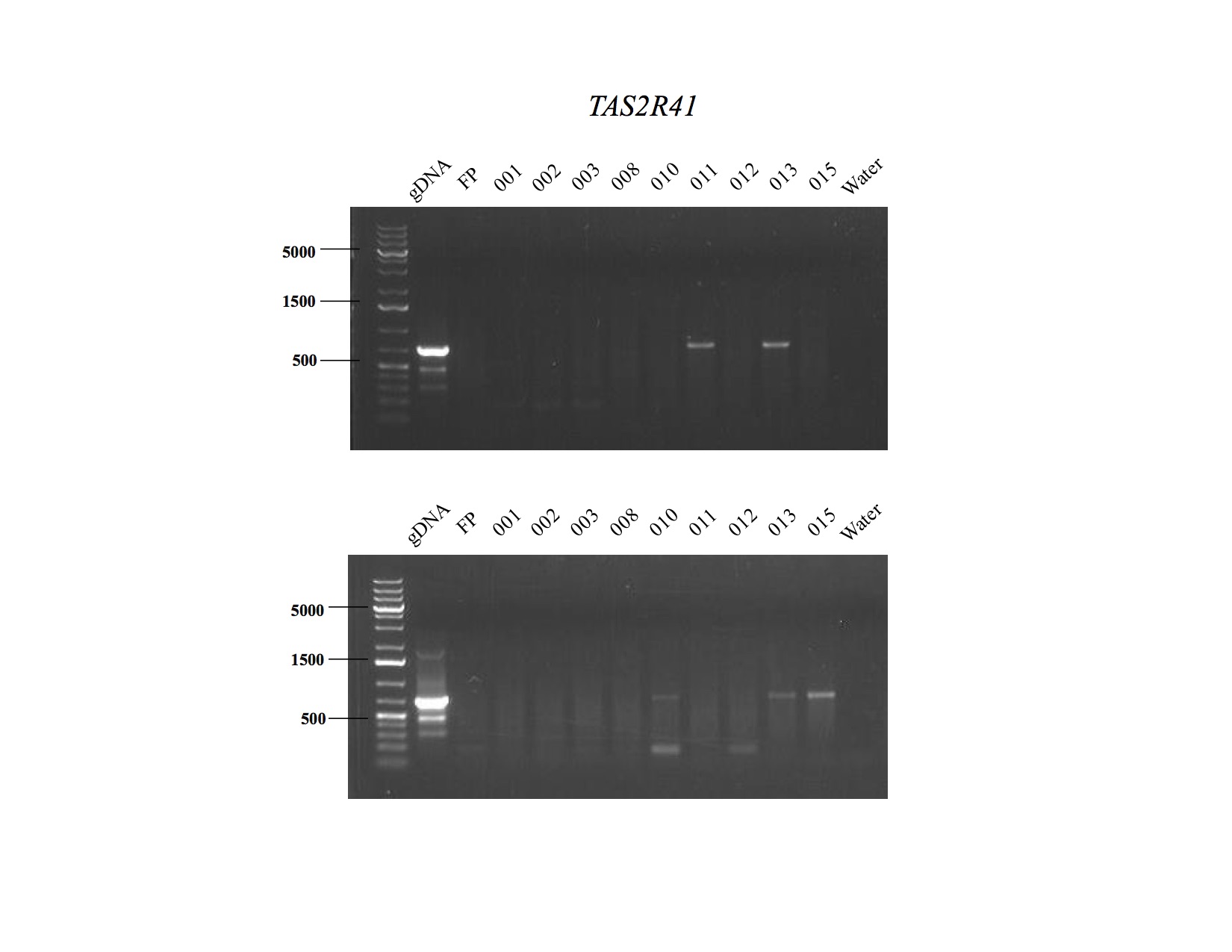

Supplement: S20 Fig — PCR was performed with genomic DNA from skin (gDNA), cDNA from fungiform papillae (FP), and cDNA from nine skin samples. Water was used as a no-template control. The expected band size is 738 bp. Multiple bands are likely because of non-specific binding. The experiment was replicated (bottom panel) because taste receptors are not abundant and can have variable results. (JPG) [file pone.0205322.s020.jpg]

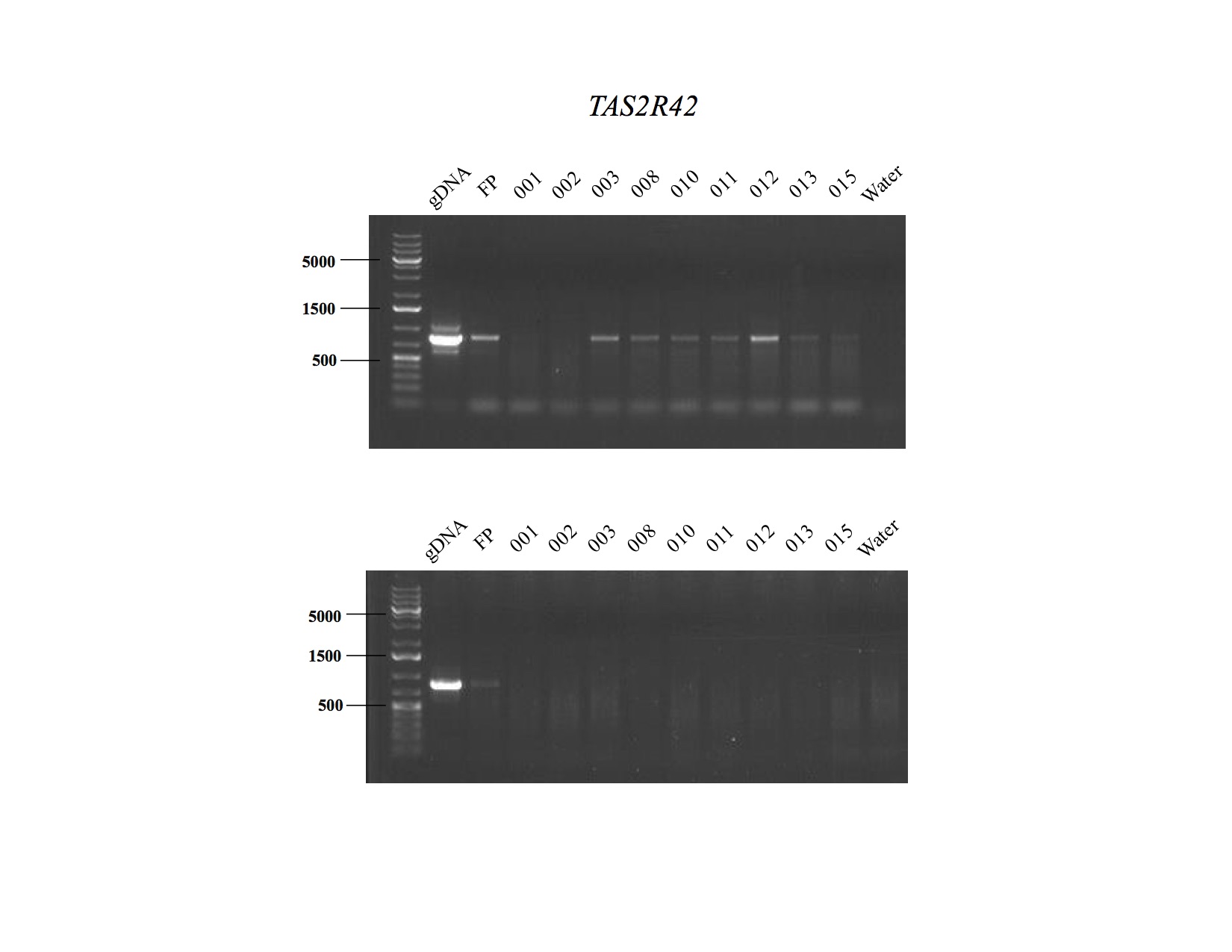

Supplement: S21 Fig — PCR was performed with genomic DNA from skin (gDNA), cDNA from fungiform papillae (FP), and cDNA from nine skin samples. Water was used as a no-template control. The expected band size is 871 bp. The experiment was replicated (bottom panel) because taste receptors are not abundant and can have variable results. (JPG) [file pone.0205322.s021.jpg]

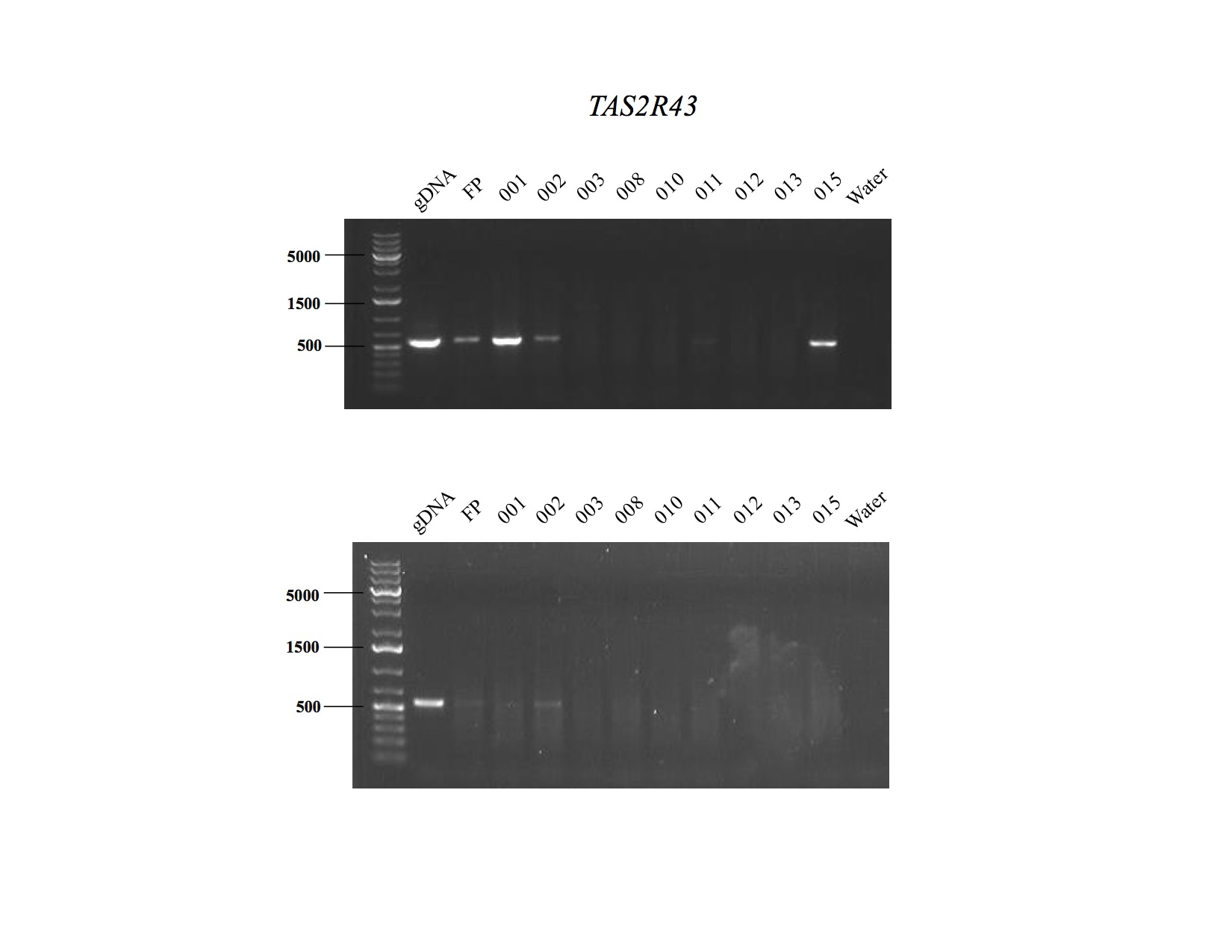

Supplement: S22 Fig — PCR was performed with genomic DNA from skin (gDNA), cDNA from fungiform papillae (FP), and cDNA from nine skin samples. Water was used as a no-template control. The expected band size is 698 bp. The experiment was replicated (bottom panel) because taste receptors are not abundant and can have variable results. (JPG) [file pone.0205322.s022.jpg]

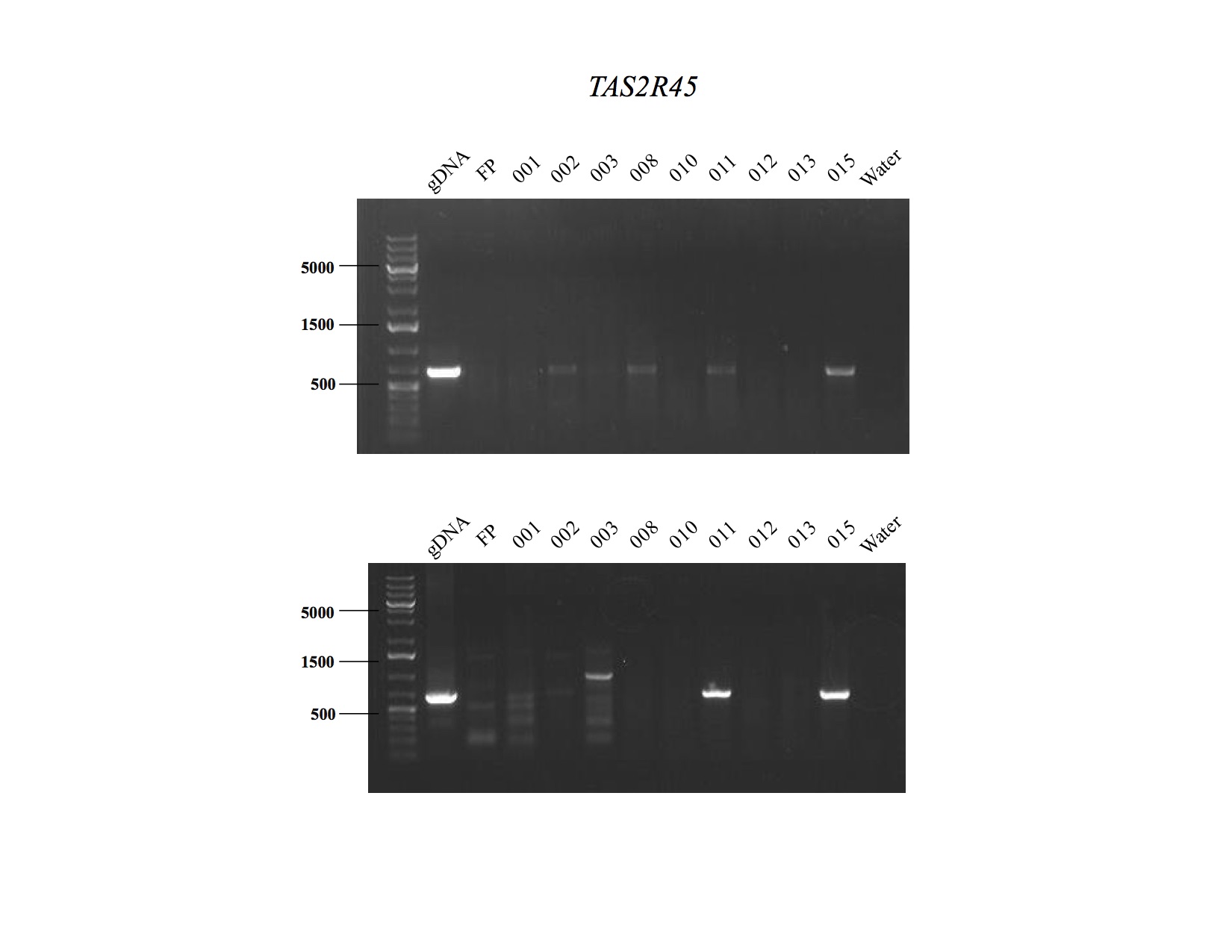

Supplement: S23 Fig — PCR was performed with genomic DNA from skin (gDNA), cDNA from fungiform papillae (FP), and cDNA from nine skin samples. Water was used as a no-template control. The expected band size is 709 bp. Multiple bands are likely because of non-specific binding. The experiment was replicated (bottom panel) because taste receptors are not abundant and can have variable results. (JPG) [file pone.0205322.s023.jpg]

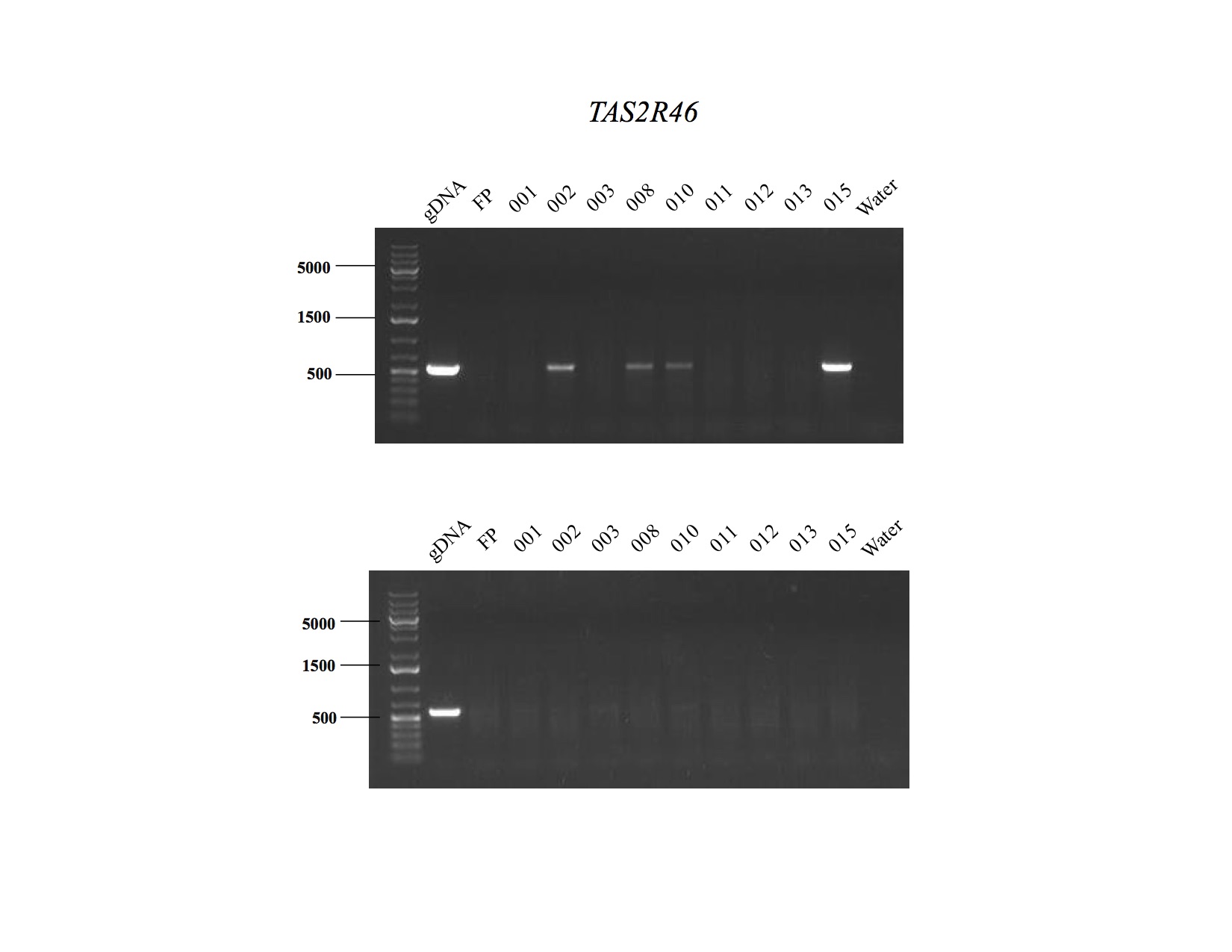

Supplement: S24 Fig — PCR was performed with genomic DNA from skin (gDNA), cDNA from fungiform papillae (FP), and cDNA from nine skin samples. Water was used as a no-template control. The expected band size is 606 bp. The experiment was replicated (bottom panel) because taste receptors are not abundant and can have variable results. (JPG) [file pone.0205322.s024.jpg]

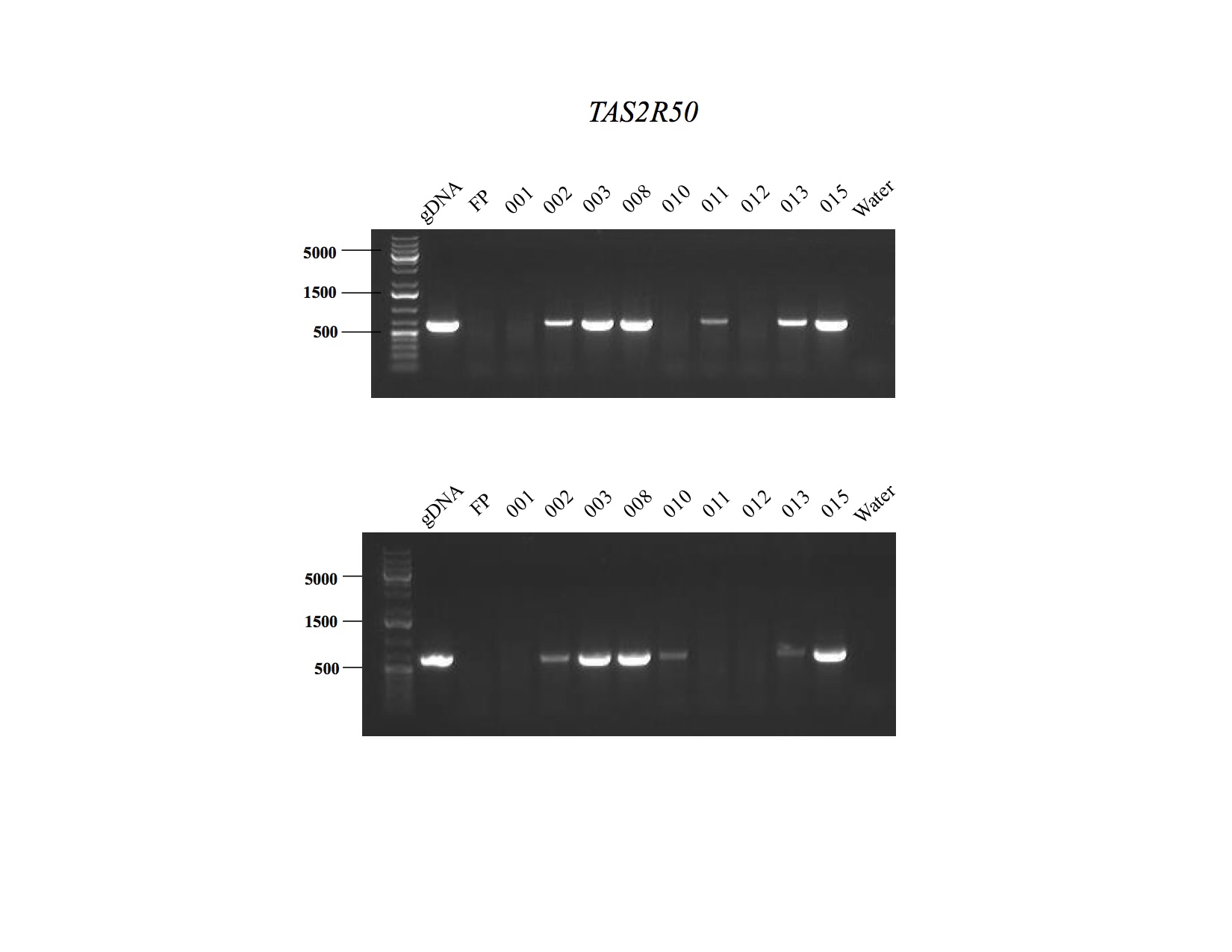

Supplement: S25 Fig — PCR was performed with genomic DNA from skin (gDNA), cDNA from fungiform papillae (FP), and cDNA from nine skin samples. Water was used as a no-template control. The expected band size is 710 bp. The experiment was replicated (bottom panel) because taste receptors are not abundant and can have variable results. (JPG) [file pone.0205322.s025.jpg]

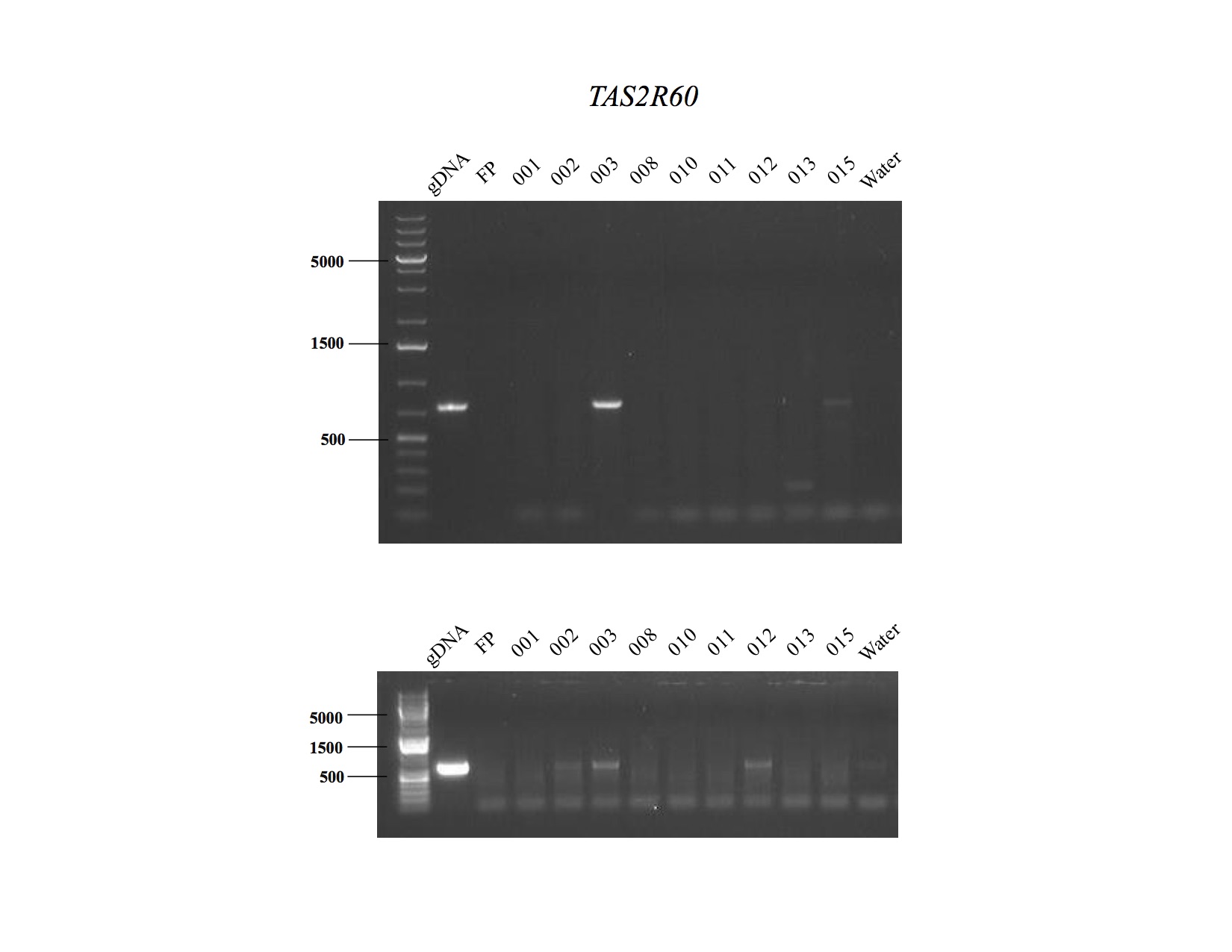

Supplement: S26 Fig — PCR was performed with genomic DNA from skin (gDNA), cDNA from fungiform papillae (FP), and cDNA from nine skin samples. Water was used as a no-template control. The expected band size is 748 bp. The experiment was replicated (bottom panel) because taste receptors are not abundant and can have variable results. (JPG) [file pone.0205322.s026.jpg]

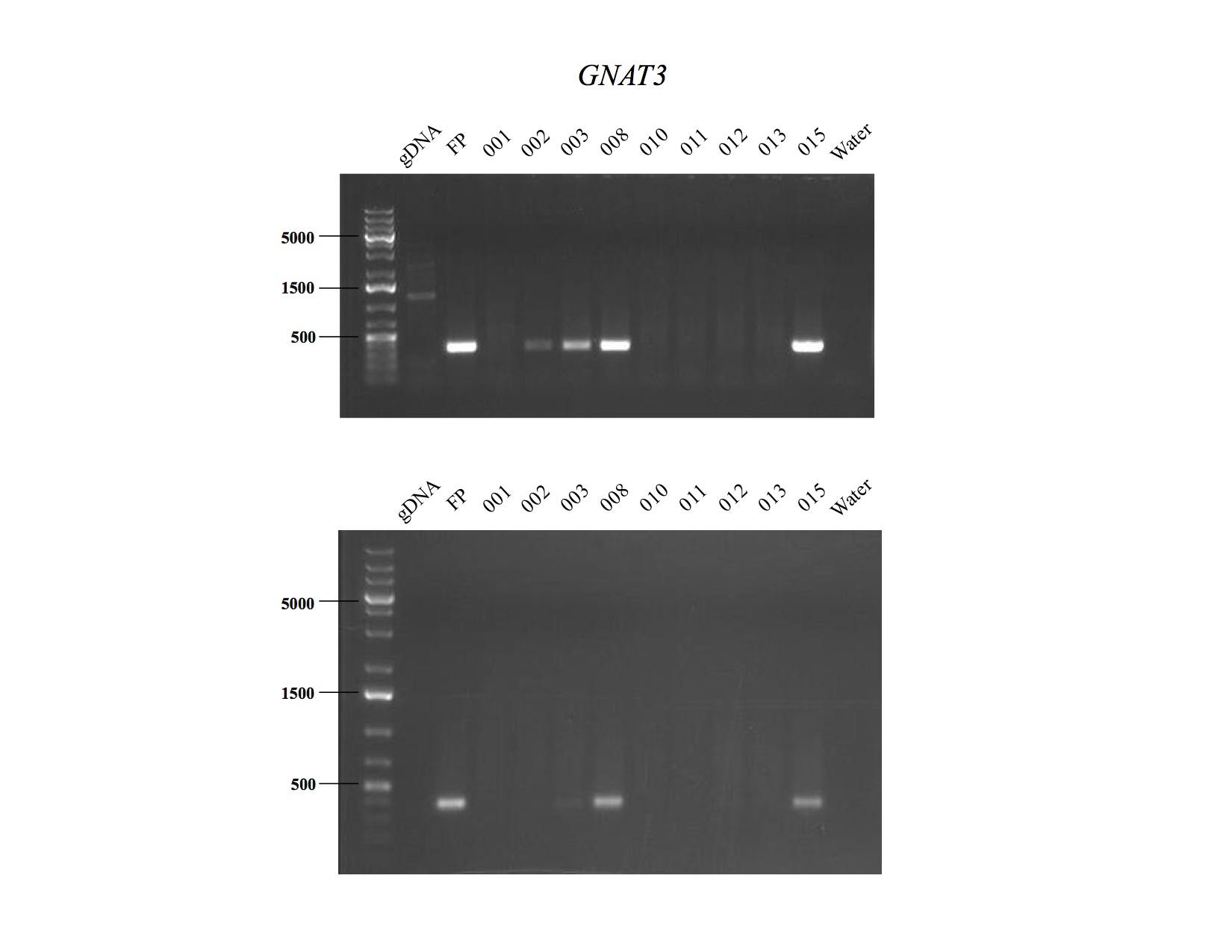

Supplement: S27 Fig — PCR was performed with genomic DNA from skin (gDNA), cDNA from fungiform papillae (FP), and cDNA from nine skin samples. Water was used as a no-template control. The primer set is intron-spanning, so there is no expected band size for genomic DNA, while there is an expected band size of 386 bp for cDNA. The experiment was replicated (bottom panel) because taste receptors are not abundant and can have variable results. (JPG) [file pone.0205322.s027.jpg]

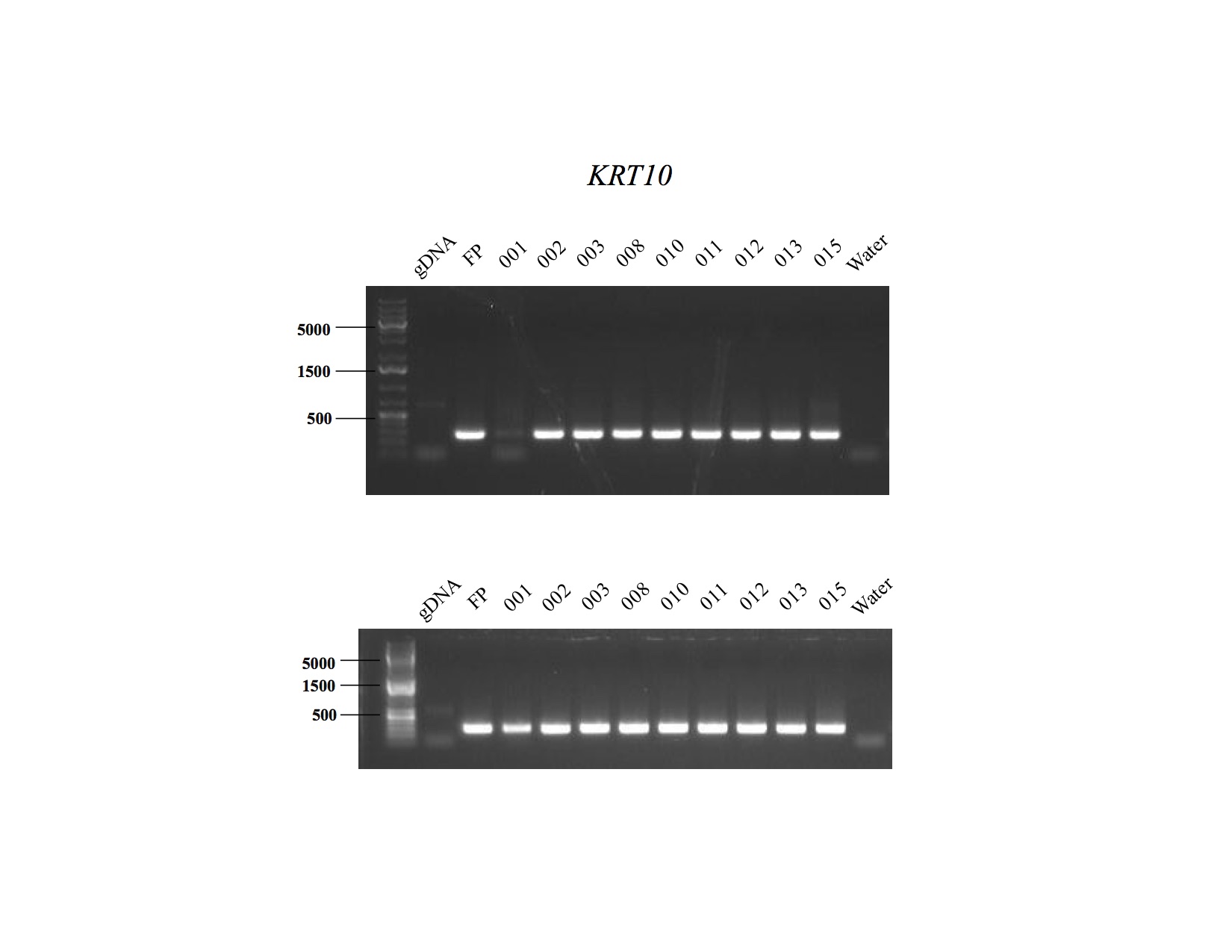

Supplement: S28 Fig — PCR was performed with genomic DNA from skin (gDNA), cDNA from fungiform papillae (FP), and cDNA from nine skin samples. Water was used as a no-template control. The primer set is intron-spanning, so there is no expected band size for genomic DNA and an expected band size of 290 bp for cDNA. The experiment was replicated (bottom panel) because taste receptors are not abundant and can have variable results. (JPG) [file pone.0205322.s028.jpg]
