# Supplementary material for: Personalized expression of bitter ‘taste’ receptors in human skin
Source: PLoS One. 2018 Oct 17;13(10):e0205322. doi: 10.1371/journal.pone.0205322 (PMC6192714; doi:10.1371/journal.pone.0205322)
Supplement: S1 Table — (PDF) [file pone.0205322.s029.pdf]

S1 Table. Sun-exposed vs. not sun-exposed

Gene	$M_{Sun\text{-}exposed}$	$M_{Not\ sun\text{-}exposed}$	*Kruskal-Wallis chi-squared*	*DF*	*P*	*Summary*
TAS2R1	0	0	0.778	1	0.3777	n.s.
TAS2R3	0.110	0.120	0.232	1	0.6302	n.s.
TAS2R4	0.554	0.596	1.604	1	0.2053	n.s.
TAS2R5	1.774	1.885	1.345	1	0.2462	n.s.
TAS2R7	0	0	3.795	1	0.0514	n.s.
TAS2R8	0	0	0.169	1	0.6808	n.s.
TAS2R9	0	0	2.885	1	0.0894	n.s.
TAS2R10	0.109	0.083	3.492	1	0.06167	n.s.
TAS2R13	0.023	0.022	0.103	1	0.7488	n.s.
TAS2R14	0.701	0.785	5.401	1	0.02012	*
TAS2R16	0	0	0.142	1	0.7059	n.s.
TAS2R19	0.184	0.186	0.055	1	0.8138	n.s.
TAS2R20	0.774	0.750	0.466	1	0.495	n.s.
TAS2R30	0.025	0.034	9.381	1	0.002192	**
TAS2R31	0.210	0.186	1.017	1	0.3132	n.s.
TAS2R38	0	0	3.166	1	0.07521	n.s.
TAS2R39	0	0	0.022	1	0.8829	n.s.
TAS2R40	0	0	0.453	1	0.501	n.s.
TAS2R41	0	0	3.662	1	0.05565	n.s.
TAS2R42	0	0	4.624	1	0.03153	*
TAS2R43	0.032	0.036	0.828	1	0.3628	n.s.
TAS2R46	0.037	0.039	0.404	1	0.5248	n.s.
TAS2R50	0.031	0.036	3.452	1	0.06316	n.s.
TAS2R60	0.046	0	66.581	1	3.359e-16	****
