# Supplementary material for: Personalized expression of bitter ‘taste’ receptors in human skin
Source: PLoS One. 2018 Oct 17;13(10):e0205322. doi: 10.1371/journal.pone.0205322 (PMC6192714; doi:10.1371/journal.pone.0205322)
Supplement: S2 Table — (PDF) [file pone.0205322.s030.pdf]

S2 Table. Male vs. female in not sun-exposed tissue

Gene	M_{Female}	M_{Male}	*Kruskal-Wallis chi-squared*	*DF*	*P*	*Summary*
TAS2R1	0	0	1.593	1	0.215	n.s.
TAS2R3	0.144	0.110	6.947	1	0.008	**
TAS2R4	0.677	0.551	10.381	1	0.001	**
TAS2R5	1.928	1.831	1.960	1	0.162	n.s.
TAS2R7	0	0	0.444	1	0.505	n.s.
TAS2R8	0	0	5.467	1	0.019	*
TAS2R9	0	0	0.079	1	0.779	n.s.
TAS2R10	0.081	0.084	0.019	1	0.891	n.s.
TAS2R13	0.023	0.022	0.628	1	0.428	n.s.
TAS2R14	0.747	0.793	0.792	1	0.374	n.s.
TAS2R16	0	0	0.839	1	0.360	n.s.
TAS2R19	0.179	0.186	0.470	1	0.493	n.s.
TAS2R20	0.741	0.742	0.007	1	0.931	n.s.
TAS2R30	0.031	0.039	2.391	1	0.122	n.s.
TAS2R31	0.180	0.191	2.307	1	0.129	n.s.
TAS2R38	0	0	0.008	1	0.929	n.s.
TAS2R39	0	0	0.904	1	0.342	n.s.
TAS2R40	0	0	0.002	1	0.966	n.s.
TAS2R41	0	0	0.056	1	0.823	n.s.
TAS2R42	0	0	0.029	1	0.865	n.s.
TAS2R43	0.038	0.035	0.965	1	0.326	n.s.
TAS2R46	0.044	0.040	0.019	1	0.890	n.s.
TAS2R50	0.033	0.037	0.911	1	0.340	n.s.
TAS2R60	0	0.014	0.007	1	0.932	n.s.
