# Supplementary material for: Personalized expression of bitter ‘taste’ receptors in human skin
Source: PLoS One. 2018 Oct 17;13(10):e0205322. doi: 10.1371/journal.pone.0205322 (PMC6192714; doi:10.1371/journal.pone.0205322)
Supplement: S3 Table — (PDF) [file pone.0205322.s031.pdf]

S3 Table. Male vs. female in sun-exposed tissue

Gene	M_{Female}	M_{Male}	*Kruskal-Wallis chi-squared*	*DF*	*P*	*Summary*
TAS2R1	0	0	0.130	1	0.718	n.s.
TAS2R3	0.088	0.111	4.172	1	0.041	*
TAS2R4	0.511	0.590	2.885	1	0.089	n.s.
TAS2R5	1.765	1.815	0.605	1	0.437	n.s.
TAS2R7	0	0	1.650	1	0.199	n.s.
TAS2R8	0	0	0.000	1	0.997	n.s.
TAS2R9	0	0	7.081	1	0.008	**
TAS2R10	0.092	0.107	3.482	1	0.062	n.s.
TAS2R13	0.020	0.024	3.182	1	0.074	n.s.
TAS2R14	0.635	0.715	7.131	1	0.008	**
TAS2R16	0	0	0.190	1	0.663	n.s.
TAS2R19	0.197	0.187	0.025	1	0.875	n.s.
TAS2R20	0.714	0.772	2.569	1	0.109	n.s.
TAS2R30	0.030	0.027	0.022	1	0.881	n.s.
TAS2R31	0.201	0.206	0.438	1	0.508	n.s.
TAS2R38	0	0	0.106	1	0.733	n.s.
TAS2R39	0	0	0.003	1	0.959	n.s.
TAS2R40	0	0	0.077	1	0.782	n.s.
TAS2R41	0	0	0.211	1	0.646	n.s.
TAS2R42	0	0	2.030	1	0.154	n.s.
TAS2R43	0.028	0.035	1.215	1	0.270	n.s.
TAS2R46	0.037	0.037	0.607	1	0.436	n.s.
TAS2R50	0.027	0.034	2.256	1	0.133	n.s.
TAS2R60	0.060	0.041	7.629	1	0.006	**
